# Superresolution imaging with optical fluctuation using speckle patterns illumination

**DOI:** 10.1038/srep16525

**Published:** 2015-11-17

**Authors:** MinKwan Kim, ChungHyun Park, Christophe Rodriguez, YongKeun Park, Yong-Hoon Cho

**Affiliations:** 1 Graduate School of Nanoscience and Technology, Korea Advanced Institute of Science and Technology, Daejeon 305-701, Republic of Korea; 2 Department of Physics, Korea Advanced Institute of Science and Technology, Daejeon 305-701, Republic of Korea; 3 KI for the NanoCentury, Korea Advanced Institute of Science and Technology, Daejeon 305-701, Republic of Korea

## Abstract

Superresolution fluorescence microscopy possesses an important role for the study of processes in biological cells with subdiffraction resolution. Recently, superresolution methods employing the emission properties of fluorophores have rapidly evolved due to their technical simplicity and direct applicability to existing microscopes. However, the application of these methods has been limited to samples labeled with fluorophores that can exhibit intrinsic emission properties at a restricted timescale, especially stochastic blinking. Here, we present a superresolution method that can be performed using general fluorophores, regardless of this intrinsic property. Utilizing speckle patterns illumination, temporal emission fluctuation of fluorophores is induced and controlled, from which a superresolution image can be obtained exploiting its statistical property. Using this method, we demonstrate, theoretically and experimentally, the capability to produce subdiffraction resolution images. A spatial resolution of 500 nm, 300 nm and 140 nm with 0.4, 0.5 and 1.4 NA objective lenses respectively was achieved in various samples with an enhancement factor of 1.6 compared to conventional fluorescence microscopy.

Fluorescence microscopy provides important functions for the study of biological processes at the cellular and subcellular levels with unprecedented molecular specificity. However, spatial resolution in conventional fluorescence microscopy is fundamentally limited by the optical diffraction barrier known as Abbe’s limit^1^. This resolution limit represents an unavoidable barrier when using this technology to investigate the structures and functions of biomolecules at sizes less than the half of a light wavelength. To overcome this diffraction barrier^2^,^3^,^4^, several forms of superresolution fluorescence microscopy techniques^5^,^6^,^7^,^8^,^9^ have been developed in recent decades. Among them, superresolution methods including photo-activated localization microscopy (PALM)^10^,^11^,^12^ and stochastic optical reconstruction microscopy (STORM)^13^,^14^, using the emission properties of fluorophores, have attracted significant interest due to their simplicity and easily applicability to existing commercial light microscopes. Despite these advantages, this technology cannot be used for superresolution in the general biological studies. This is because it requires complex equipment and modification of fluorophore functions to assign and control the emission properties of fluorophores^15^,^16^. Lately, as a means to alleviate these requirements, superresolution optical fluctuation imaging (SOFI)^17^ was introduced, which is based on statistical analysis of fluctuation caused by intrinsic blinking in fluorophores. Without requiring complex equipment, SOFI can provide a superresolution image with a high signal-to-noise ratio using temporally fluctuating signals of fluorophores and cumulant analysis which is a form of statistical analysis related to correlation. Moreover, SOFI has shown potential for use with conventional microscopes.

However, since SOFI requires two important properties known as blinking and correlation, to obtain superresolution images, SOFI has been only applicable for samples labeled with quantum dots and organic and protein fluorophores^18^ that have intrinsic blinking characteristic within a timescale limited by the imaging system. These requirements restrict direct application of SOFI to study of biological processes since many common fluorophores conjugated to biological samples have short blinking timescales. Several approaches^19^,^20^ have been introduced to overcome these limitations of the SOFI technique. Even though these methods extended the selectivity of fluorophores in SOFI, they still face limitations similar to those of SOFI. Thus, to overcome the limitations of SOFI completely, it is important that the optical fluctuation caused by the intrinsic blinking of fluorophores in SOFI is replaced by controllable fluctuation that is directly and simply induced by an external method, such as random patterns illumination, and that is unaffected by the type of fluorophore.

Here, we propose a new approach combining SOFI with speckle patterns illumination (S-SOFI) to generate illumination-induced optical fluctuation. In this approach, varying the speckle patterns illumination induces temporal fluctuation of fluorophore emission signals. These speckle-induced flickering signals from the fluorophores are analyzed and reconstructed using a SOFI algorithm to generate suprresolution images. Since the speckle pattern employed as the random pattern has many interesting statistical characteristics^21^, speckle patterns have already been used in several superresolution methods^22^,^23^ combining nonlinear analysis to extract high spatial frequency components of samples. However, nonlinear analysis is intractable and requires a great deal of computational time to get a superresolution image. Therefore, in this paper, we develop S-SOFI microscopy and demonstrate that not only does it give direct superresolution images (without nonlinear analysis), but also overcomes previous limitations of the SOFI method.

## Principles

**Figure 1:**
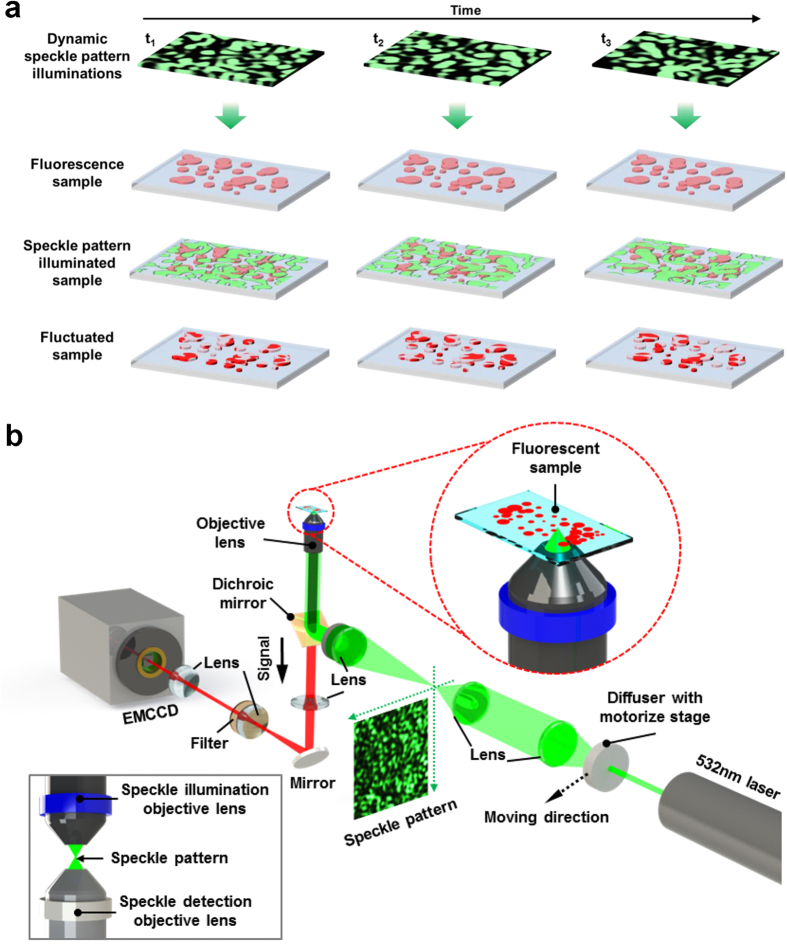
Schematic diagram of S-SOFI concept and experimental setup of S-SOFI. (**a**) Dynamic speckle patterns, which were made by moving the disordered medium, illuminate the sample labelled with fluorophores. These speckle patterns induce fluctuation in the emission of the fluorophores. (**b**) A disordered medium is set at the illumination part of a conventional inverted microscope with a motorize stage, to make speckle patterns and correlation between speckle patterns. To use modified correlation, this setup was designed such that speckle patterns were demagnified by the objective lens. The inset of (**b**), inside the gray-line box, shows the experimental setup modified by combining the illumination objective lens to measure the speckle pattern itself, using a detection objective lens of higher NA than for the illumination, to prevent loss of speckle pattern information.

The principle of S-SOFI is illustrated in Fig. 1a. Since speckle pattern has spots with random shapes, fluorophore probes of target sample can be randomly and temporally fluctuated when time-varying speckle patterns are used as illumination. Under fluctuating illumination, the intensity of fluorescence signals from N single emitters with specific distribution located at position ***r***_*k*_ at time t is described by


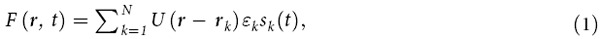


where *U*(∙) denotes the diffraction-limited point spread function in the case of an incoherent source, *ε*_*k*_ denotes molecular brightness and *s*_*k*_(*t*) represents fluctuation signals at time *t*. In the S-SOFI method, *s*_*k*_(*t*) term which represents fluctuation that does not come from intrinsic fluctuation of fluorophores, but from induced fluctuation using speckle patterns illumination. This is unlike original SOFI. As mentioned in the previous section, using the SOFI method requires correlation analysis of fluctuation signals in order to construct high resolution images; therefore, we have to consider not only fluctuation of fluorophores induced by varying speckle patterns illumination, but also their correlation.

Among many interesting properties of speckle patterns including correlation^24^,^25^,^26^,^27^ and memory effect^28^,^29^, speckle patterns correlation (The key property in our approach) was experimentally and theoretically demonstrated by Shapiro^24^ and Stephen *et al.*^25^. This is a type of spatial correlation defined as a function of the relative position between speckle patterns. Therefore, to convert this spatial correlation to temporal correlation, we need to control the lateral displacement between the disordered medium and the source at submicron scale, with time. This is determined by the minimum value between the mean free path l of a disordered medium and the incident light wavelength λ (details of speckle patterns correlation are provided the Supplementary Information). It is a challenge for S-SOFI to displace the disordered medium at submicron scale in order to employ this correlation property. However, interestingly, this correlation can be modified using a linear optics system, according to Goodman^21^. For example, speckle patterns may be illuminated by an optical system with demagnification *M_d_* = *1/M*, then correlation can be interpreted approximately as a delta function. This means that correlation of speckle patterns passed through a linear optical system with demagnification, can be modified into the point spread function (PSF) of the optical system (detailed description in Supplementary Information). Therefore, it can be given by


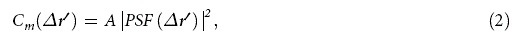


where *C*_*m*_(∙) denotes the modified spatial correlation, *A* denotes a constant of modified spatial correlation, *PSF*(∙) denotes a diffraction-limited PSF defined in case of coherent source, ***r ′*** denotes a rescaled coordinate with demagnification and *Δ**r ′*** denotes a rescaled relative position. Thus, since equation (2) implies that the modified spatial correlation function can survive at a longer range than l and λ, the restriction of control range of the lateral displacement for correlation can be relaxed enough to employ speckle patterns illumination.

## Simulation with an analytic model and a real speckle pattern

The experimental setup used to apply S-SOFI for commercial optical microscopes is shown in Fig. 1b. To make speckle patterns with fluctuation and correlation, a diffuser (DG20-1500-MD, Thorlab, USA) used as a disordered medium, was installed with a motorized stage (LNR50S, Thorlab, USA) for translational displacement at the part of illumination of a conventional inverted light microscope. These speckle patterns can be temporally fluctuated by shifting the diffuser using the motorized stage. Using these varying speckle patterns as illumination, the labeling fluorophores of the sample are excited and the emissions temporally fluctuate. Finally, this fluctuating fluorescence signal is measured as fluorescence intensity images by an electron-multiplying charge coupled device (EMCCD, Andor iXon DU 897D, Belfast, UK) over time (details of the experimental setup in Methods). The measured fluorescence images are then analyzed using the SOFI algorithm.

To demonstrate the improved resolution of S-SOFI, we should compare a reconstructed image with a wide field image obtained under uniform illumination. For a fair comparison, the average of speckle patterns illumination used for reconstruction is a reasonable substitute to uniform illumination (detailed description of uniform illumination in Supplementary Information).

We first performed numerical simulations to evaluate the performance of S-SOFI. Simulated images of a virtual sample containing two fluorophores with interdistance ***a*** was reconstructed via an analytic model and experimental simulation, under the assumption that the numerical aperture (NA) of an objective lens for illumination is 0.5, and that the wavelength of light is 532 nm. The second order cumulant function *G*_*2*_(***r**, τ*) in the case of a virtual sample containing two fluorophores, based on equations (1), is given by


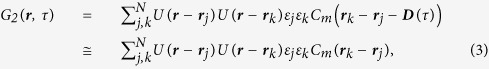


where *τ* is the time lag between each frame, *C*_*m*_ is the correlation made by speckle patterns and ***D***(*τ*) is the step size of the motorized stage rescaled in relation to optical magnification. Since ***D***(*τ*) can be neglected in the calculation when it is rescaled according to optical magnification, and the step size of the motorized stage is small enough, we can get a simple equation, such as in equation (3) (details of ***D***(*τ*) in Supplementary Information). Based on this equation, the S-SOFI image of an analytic model is calculated (detailed description in Supplementary Information).

**Figure 2:**
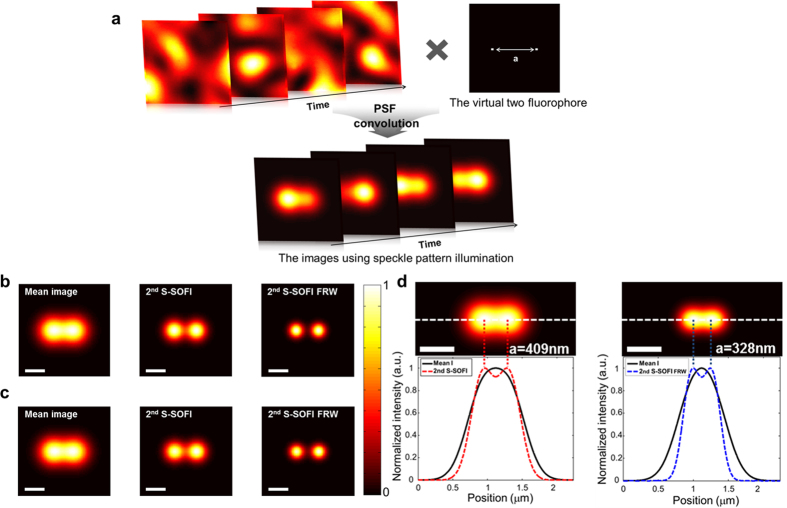
Schematic steps for experimental simulation and results. (**a**) Each of 300 correlated speckle patterns (motorized stage step = 4 μm, objective lens NA = 0.5) provided by the experimental setup is multiplied with the virtual sample containing two fluorophores. Finally, the images produced with speckle patterns illumination were obtained by convolution using a detection point spread function with NA = 0.5. (**b**) Images of two fluorophores ***a*** = 544 nm apart were obtained by analytic model simulation. (**c**) Images of the same fluorophores as (**b**) were gotten using experimental simulation. Mean images were produced by averaging of 300 speckle patterns illumination images. The 2^nd^ S-SOFI image was reconstructed using the 2^nd^ order SOFI algorithm, and the 2^nd^ S-SOFI FRW image was gotten from the Fourier reweighting method. (**d**,**e**) The images show the resolution limits of 2^nd^ SOFI, and 2^nd^ SOFI with FRW, employing the criterion of the Abbe limit. The color bar represents normalized intensity. The scale bars in all figures indicate 500 nm

Experimental simulation was performed using the correlated speckle patterns measured using the experimental setup, combined with the illumination objective lens (inset of Fig. 1b). A pixel size of the EMCCD corresponds to 32 nm at the sample plane at 500× magnification (detailed experimental setup in Methods and Supplementary information). Virtual sample images illuminated by 300 correlated speckle patterns, were calculated through convolution of the measured speckle patterns and virtual sample as illustrated Fig. 2a. For comparison of the two simulations, the images of a virtual sample containing two fluorophores, and an interdistance of ***a*** = 544 nm (near the diffraction-limited distance), were simulated with each method under the same simulation conditions. In addition, the Fourier reweighting (FRW) method introduced by Dertinger *et al.*^30^ was applied to both simulations to improve the resolution enhancement of SOFI. The FRW approach allows deduction of the maximum spatial frequency physically accessible from measured images, using a simple reweighting for the optical transfer function (OTF) (detailed description of FRW in Methods and Supplementary Information). Reconstructing images of S-SOFI and FRW through the analytic model and experimental simulation, superreoslution images were obtained as shown in Fig. 2b,c, respectively. As illustrated in Fig. 2b,c, the images from the sample containing two fluorophores show similar results. These results mean that analytic model is in good agreement with the real measurement of S-SOFI. Therefore, the analytic model can be used to estimate the S-SOFI resolution. To define the resolution enhancement of our method, the analytic model was used to calculate a matrix with pixel size of 1 nm. Using the analytic model and the criterion defining Abbe’s limit, resolution enhancement was evaluated as follows. The second order S-SOFI (2^nd^ S-SOFI) showed 1.3× enhancement (Fig. 2d) and the second order S-SOFI with FRW (2^nd^ S-SOFI with FRW) showed 1.6× enhancement (Fig. 2e).

## Experimental imaging

**Figure 3:**
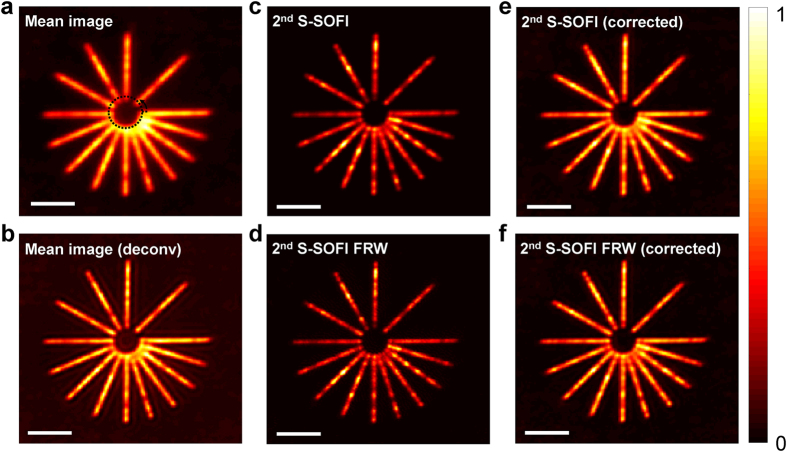
Experimental S-SOFI data from the radial fluorescent nanopattern. The sample was made using E-beam lithography from hydrogen silsesquioxane (HSQ) mixed with Rhodamine 6G fluorescent dye. It was illuminated by 700 correlated speckle patterns with a motorized stage with step size of 6 μm and an objective lens NA = 0.5. (**a**) Mean image from averaging 700 images, (**b**) The deconvolution of the image (**a**) with PSF, (**c**) 2^nd^ S-SOFI image reconstructed by the 2^nd^ order SOFI algorithm from 700 images, (**d**) 2^nd^ S-SOFI FRW image reconstructed by applying the Fourier reweighting method to 2^nd^ S-SOFI images. (**e**,**f**) The images correcting the distortion of amplified molecular brightness from (**c,d**). The color bar represents normalized intensity. The scale bars in all figures indicate 5 μm.

**Figure 4:**
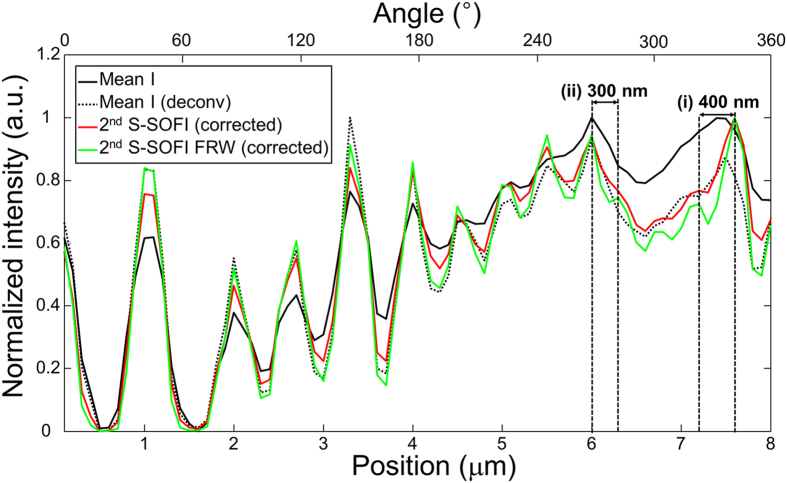
Resolution enhancement of S-SOFI. The cross-sectional line profiles along the dashed line with black circle in Fig. 3a. And same line profiles were taken from Fig. 3b,e,f. Note that the 2^nd^ S-SOFI image can resolve two lines that are ~400 nm apart, as shown in (i), and the 2^nd^ S-SOFI with FRW image can resolve two lines that are ~300 nm apart, as shown in (ii).

S-SOFI was then validated using experimental images of radial fluorescent nanopatterns obtained from the experimental setup in Fig. 1b, using an objective lens with NA = 0.5. The radial fluorescent nanopattern was made by E-beam lithography using a mixture of Rhodamine 6G fluorescent dyes and hydrogen silsesquioxane (HSQ) which formed a negative e-beam resist on the glass substrate. According to simulation prediction and Nyquist sampling theory, the imaging pixel size was adjusted to 100 nm through 160× magnification for detection (details of samples are given in Methods). Sample images (as illustrated in Supplementary Movie 1) were measured with 700 correlated speckle patterns used as illumination (exposure time = 60 ms), and processed using the SOFI algorithm and FRW method. The 2^nd^ S-SOFI image (Fig. 3c) and 2^nd^ S-SOFI with FRW (Fig. 3d) exhibited better resolution image compared with the conventional wide field image (Fig. 3a). However, contrast of 2^nd^ S-SOFI image (Fig. 3c) shows distortion compared to that of wide field image simulation results in previous section. This contrast distortion comes from the amplified molecular brightness *ε*, which is squared in equation (3). To remove this unwanted side effect, the amplified molecular brightness is corrected by simple linearization using deconvolution. Similar technique have been used to correct amplified molecular brightness in high order SOFI^31^. As shown in correction images (Fig. 3e,f), the contrast distortion almost disappears (detail description of contrast distortion correction in Methods). The line profiles of each image (dashed lines with black circles in Fig. 3a) show that the 2^nd^ S-SOFI (Fig. 3e), and 2^nd^ S-SOFI with FRW (Fig. 3f), can resolve two adjacent lines of the nanopattern that are ~400 and ~300 nm apart (Fig. 4 (i) and (ii), respectively). These results are the almost the same as those obtained from the analytic model. With this result, we verified that our analytic model is able to describe S-SOFI with good accuracy.

**Figure 5:**
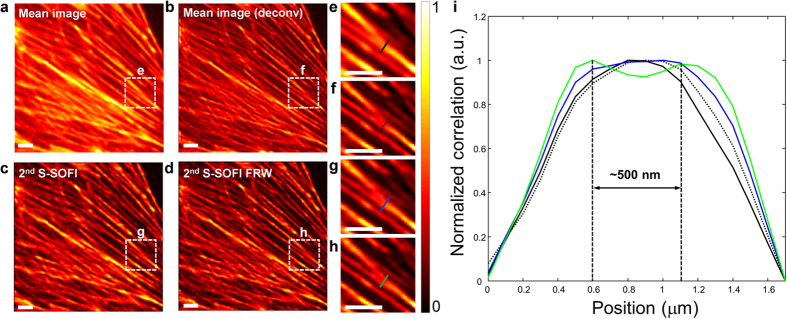
Experimental S-SOFI data of the biological sample obtained with a low NA (0.4) objective lens and the cross sectional intensity line profiles. The sample is an actin filament of bovine pulmonary artery endothelial cells (BPAEC) from a biological test slide (FluoCells® prepared slide #2 (F14781), Molecular probes, Eugene, OR, USA) labeled with Texas Red (T7471, Lifetechnologies, USA). This sample images were obtained by illumination using 700 correlated speckle patterns with motorized stage with step size of 2 μm and objective lens NA = 0.4. (**a**) Mean image from averaging 700 images, (**b**) The deconvolved image of (**a**), (**c**) 2^nd^ S-SOFI image processed by 2^nd^ SOFI algorithm and correction, (**d**) 2^nd^ S-SOFI FRW reconstructed by applying the Fourier reweighting method to 2^nd^ SOFI images. (**e**–**h**) They are expanded images of the regions within the dashed line boxes. The color bar represents normalized intensity. (**i**) The cross-sectional intensity line profiles were taken from the colored dashed lines in (**e**–**h**). Black solid line indicates wide field image. Black dot line indicates deconvolution image. And each blue, green solid line indicates 2^nd^ SOFI and 2^nd^ SOFI FRW image, respectively. All the scale bars indicate 5 μm.

**Figure 6:**
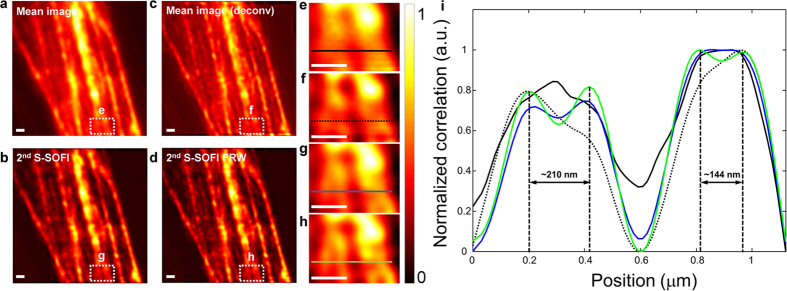
Experimental S-SOFI data of the biological sample obtained with a high NA (1.4) objective lens and the cross sectional intensity line profiles. 700 frames of image of sample were obtained with motorized stage wih step size of 7 μm and objective lens NA = 1.4. (**a**) Mean image from averaging 700 images, (**b**) The deconvolved image of (**a,c**) 2^nd^ S-SOFI image processed by 2^nd^ SOFI algorithm and brightness correction, (**d**) 2^nd^ S-SOFI FRW reconstructed by applying the Fourier reweighting method to 2^nd^ SOFI images. (**e**–**h**) They are expanded images of the regions within the dashed line boxes. The color bar represents normalized intensity. (**i**) The cross-sectional intensity line profiles were taken from the colored dashed lines in (**e**–**h**). Black solid line indicates wide field image. Black dot line indicates deconvolution image. And each blue, green solid line indicates 2^nd^ SOFI and 2^nd^ SOFI FRW image, respectively. All the scale bars indicate 500 nm.

Finally, S-SOFI was applied to image a biological cell. The samples were illuminated with 700 correlated speckle patterns using a microscope objective lenses with NA = 0.4 and 1.4. To precisely verify the enhanced resolution, a single pixel size in the EMCCD corresponds to 100 nm (0.4 NA) and 16 nm (1.4 NA) in the sample plane due to a detection magnification 160× and 1000×, respectively. The biological sample used, was fixed actin filaments in bovine pulmonary artery endothelial cells (BPAEC) on biological test slide (FluoCells® prepared slide#2 (F14781), Molecular probes, Eugene, OR, USA), labeled with Texas Red®-X Phalloidin (T7471, Lifetechnologies, USA). Seven hundred images provided by various speckle pattern illuminations (exposure time = 100 ms (0.4 NA) and 200 ms (1.4 NA)) were processed using SOFI, the FRW algorithm, and molecular brightness correction. As clearly shown in Figs 5c,d and 6c,d, the 2^nd^ S-SOFI image, and 2^nd^ S-SOFI with FRW, showed significant improvements in spatial resolution compared to the conventional wide field image and deconvolution image (Figs 5a,b and 6a,b). As shown in Figs 5i and 6i, this improvement in resolution can also be observed in the line profiles depicted with dashed lines in Figs 5e–h and 6e–he–h, respectively.

## Discussion

Utilizing controllable temporal optical fluctuation generated from speckle patterns, we developed S-SOFI for superresolution based on the induced fluctuation and correlation with speckle patterns illuminations. The capability of S-SOFI was demonstrated using analytic calculation, simulation, experimental methods. The reconstructed images could provide at least 1.6× resolution enhancement (2^nd^ S-SOFI with FRW) compared with the conventional wide field microscopy. In this article, S-SOFI was demonstrated using a far-field speckle pattern at the diffraction-limited size of the optical system. This diffraction-limited speckle pattern has been regarded as unable to provide subdiffraction resolution images since it makes it impossible for the fluorophores to have totally independent fluctuation. Even though the resolution enhancement of S-SOFI cannot reach the root of the cumulant order unlike original SOFI because of the cross correlation between fluorophores comes from the diffraction-limited size of the speckle patterns (detail description in Supplementary), we arrived at the surprising conclusion that S-SOFI method can achieve superresolution contrary to general expectation.

The S-SOFI method includes some limitations, such as low resolution enhancement and the need for many image frames for superresolution imaging. The demonstrated 1.6× resolution enhancement of S-SOFI is relatively low compared to other superresolution methods that utilize the emission properties of fluorophores. However, the resolution enhancement could be increased using speckle patterns at subdiffraction size (e.g., near field speckle pattern) since the enhancement of S-SOFI resolution depends the size of speckle patterns, which can also determine the magnitude of spatial correlation. Also, the number of image frames required in the imaging process could be reduced by changing the temporal correlation length, which may be controlled by adjusting the step size of the motorized stage unlike intrinsic blinking of fluorophores. However, when changing the step size of the motorized stage to control the temporal correlation, we have to consider ***D***(*τ*) in Equation (3), since it is not small enough to neglect. Thus, large ***D***(*τ*) can bring about considerable distortion during resolution enhancement of S-SOFI. To remove this effect, we applied zero time lag (*τ* = 0) approach in the SOFI algorithm^17^,^30^. Because this approach means that image fluctuations are analyzed by the SOFI algorithm without time shifting, the step size of motorized stage does not affect S-SOFI in equation (3). Applying this approach, S-SOFI is able not only to reduce the number of images needed for S-SOFI, but also to manipulate the displacement of the disordered medium without restriction. This implies that random speckle patterns, without control of step size of the motorized stage, could be used for S-SOFI. Although we showed S-SOFI results using many image frames in this article, we can demonstrate that it is possible to obtain similar results using fewer image frames by applying correlation control (details of correlation control, and the demonstration, are given in the Supplementary Information). However, for making controllable fluctuation and correlation, S-SOFI still requires additional equipment including a diffuser and a motorized stage. While this requirement limits the applications of S-SOFI, it enables the use of any sample without any concern for fluorophore type. This is because fluctuation and correlation of the fluorophore is induced and controlled by this external equipment. Furthermore, it is easier and simpler to install in a conventional light microscope compared to other superresolution methods.

In conclusion, S-SOFI has many advantages for general microscopic imaging applications. Because controllable optical fluctuation and correlation are used in this method, it can overcome the limitations of SOFI caused by dependence on fluorophore blinking. Because SOFI analysis can extract high spatial frequency components from such fluctuation, this method can give superresolution images without nonlinear analysis. Furthermore, the instruments required for S-SOFI imaging can easily be combined with existing microscopes since the only additional parts required, compared to conventional microscopy, are the disordered medium and motorize stage. This method could be extended to non-fluorescence microscopy because the fluctuation property in this method comes from illumination, not from blinking of a sample. Speckle distortion, which is concurrent with shifts in the speckle pattern^32^ and is ignored to simplify of analytic model, as well as near-field speckle pattern illumination can lead to further reduction of correlation length^33^,^34^,^35^. As a result, it is possible to increase the enhancement of resolution using higher order correlation. Not only speckle patterns used in our approach, but any illumination pattern with fluctuation and correlation, could be also used with this method.

## Methods

### Experimental setup for measuring speckle patterns

To illuminate and detect speckle patterns, a conventional inverted microscope (IX71, Olympus, USA) was combined with a conventional upright microscope (BXFM, Olympus, USA). The conventional inverted microscope was used to detect the speckle patterns, and the conventional upright microscope was used to illuminate the sample with the speckle patterns (inset of Fig. 1b). To prevent loss of spatial frequency information from the speckle pattern, higher NA objective lenses (NA = 0.8) were employed for detection, than for illumination (NA = 0.5). To make a correlated speckle pattern, the illumination part was combined with a diffuser (DG20-1500-MD, Thorlab, USA) and a motorize stage (LNR50S, Thorlab, USA). Light from a 532 nm laser (500 mW, Shanghai Dream Laser, China) illuminated the diffuser and these emerging beams from the diffuser generated correlated speckle patterns as the diffuser was moved on the motorized stage. The beams with the correlated speckle patterns propagated to the illumination objective lens. The speckle patterns were demagnified during propagation through the illumination objective. In order to use the modified correlation, we illuminated the beam with the demagnified speckle pattern through the detection objective lens and the 4f system. Finally, the speckle pattern is detected using the EMCCD (Andor iXon DU 897D, Belfast, UK), which is synchronized with the motorized stage translation diffuser using software. In this experiment, a single pixel in the EMCCD corresponds to 32 nm in the sample plane due to a lateral magnification of 500×. Thus, the speckle images are oversampled beyond the Nyquist theorem with the intention to achieve the resolution enhancement expected from the analytical model. Additionally, it provides a chance for a clear estimation of the S-SOFI resolution with an accuracy of 32 nm. A detailed figure of the experiment setup is provided in Supplementary Fig. S2.

### Experimental setup for measuring fluorescence samples

The setup for measurement of fluorescence samples was almost same as that explained in the previous section, except that the conventional microscope used for illumination was removed and that role is performed by a detection objective lens (shown in Fig. 1b). The objective lenses with NA = 0.5, NA = 0.4 and 1.4 were used for experiment of the radial fluorescent nanopattern and biological sample, respectively. The beams from the diffuser, of the correlated speckle patterns, illuminate the sample using the 4f system and a dichroic mirror (Di02-R532-25 × 36, Semrock, USA). During the propagation, speckle patterns were demagnified by the objective lens in order to use modified correlation. The illumination induced fluctuating signals from the fluorophore, which were filtered using the dichroic mirror and long-pass filter (BLP01-532R-25, Semrock, USA), and then measured using the EMCCD.

### Radial fluorescent nanopattern

The radial fluorescent nanopattern was made as follows. First, hydrogen silsesquioxane (HSQ) (Negative E-beam resist, Dow Corning Co. FOX-16, USA) was mixed with Rhodamine 6G (R1427, Sigma-Aldrich, USA). Second, this mixture was spin-coated onto at glass substrate followed by soft baking. Then the radial nanopattern was patterned on the glass substrate using E-beam lithography. Third, to remove regions other the desired pattern, the sample was developed. Finally, we achieved radial nanopattern including Rhodamine 6G. The scanning electron microscopy (SEM) image is given in Supplementary Fig. S11.

### Fourier reweighting method

The Fourier reweighting (FRW) method can improve resolution enhancement of SOFI. According to the FRW, using a simple reweighting for the optical transfer function (OTF), resolution enhancement of SOFI can be improved even more (e.g., root (2) → 2). Mathematical expression of FRW is given by


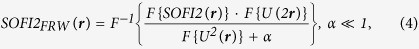


where *SOFI2*(***r***) is the 2^nd^ SOFI image obtained by S-SOFI, *SOFI2*_*FRW*_(***r***) is the 2^nd^ SOFI FRW image obtained by applying the FRW method to the 2^nd^ SOFI image, and *F*{∙}*, F*^*−1*^{∙} are the Fourier transform and inverse Fourier transform, respectively. Here, *U*(∙) is the diffraction-limited point spread function (PSF) of the optical system for the case of an incoherent source, α is a damping factor to prevent from divergence. The 2^nd^ SOFI FRW image in the experimental section was obtained using this equation.

### Correction of molecular brightness

The contrast distortion of S-SOFI image occurs by amplified molecular brightness during SOFI algorithm, as shown in equation (3). In case of simulation, because molecular brightness of fluorophores is same, this factor cannot affect contrast distortion. But, in case of real experiment, fluorophores with unequal molecular brightness distort the contrast of final image. To remove this distortion, we adopt simple linearization using deconvolution method, which consists of three steps after SOFI algorithm. First, 2^nd^ SOFI image is deconvolved with PSF using a Lucy-Richardson algorithm. Second, the deconvolved image takes the square root to linearization of molecular brightness. Finally, the image is reconvolved with PSF. From this progress and equation (3), the corrected equation is mathematically given by


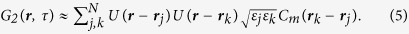


Therefore, amplified molecular brightness is corrected by simple linearization. The corrected image in the experimental section was obtained using this process.

## Additional Information

**How to cite this article**: Kim, M.K. *et al.* Superresolution imaging with optical fluctuation using speckle patterns illumination. *Sci. Rep.* **5**, 16525; doi: 10.1038/srep16525 (2015).

## Supplementary Material

Supplementary Information

Supplementary movie 1

## References

[b1] Abbe E. Conributions to the theory of the microscope and the microscopic perception (translated from German). Archiv für mikroskopische Anatomie 9, 413–418 (1873).

[b2] Huang B., Babcock H. & Zhuang X. Breaking the diffraction barrier: super-resolution imaging of cells. Cell 143, 1047–1058 (2010). 21168201 10.1016/j.cell.2010.12.002 PMC3272504

[b3] Fernandez-Suarez M. & Ting A. Y. Fluorescent probes for super-resolution imaging in living cells. Nat. Rev. Mol. Cell Biol. 9, 929–943 (2008). 19002208 10.1038/nrm2531

[b4] Hell S. W. Far-field optical nanoscopy. Science 316, 1153–1158 (2007). 17525330 10.1126/science.1137395

[b5] Hell S. W. & Wichmann J. Breaking the Diffraction Resolution Limit by Stimulated-Emission: Stimulated-Emission-Depletion Fluorescence Microscopy. Opt. Lett. 19, 780–782 (1994). 19844443 10.1364/ol.19.000780

[b6] Klar T. A. & Hell S. W. Subdiffraction resolution in far-field fluorescence microscopy. Opt. Lett. 24, 954–956 (1999). 18073907 10.1364/ol.24.000954

[b7] Hell S. W. & Kroug M. Ground-State-Depletion Fluorescence Microscopy - a Concept for Breaking the Diffraction Resolution Limit. Appl. Phys. B 60, 495–497 (1995).

[b8] Heintzmann R., Jovin T. M. & Cremer C. Saturated patterned excitation microscopy - a concept for optical resolution improvement. J. Opt. Soc. Am. A 19, 1599–1609 (2002). 10.1364/josaa.19.001599 12152701

[b9] Gustafsson M. G. Nonlinear structured-illumination microscopy: wide-field fluorescence imaging with theoretically unlimited resolution. Proc. Natl. Acad. Sci. USA 102, 13081–13086 (2005). 16141335 10.1073/pnas.0406877102 PMC1201569

[b10] Betzig E. *et al.* Imaging intracellular fluorescent proteins at nanometer resolution. Science 313, 1642–1645 (2006). 16902090 10.1126/science.1127344

[b11] Hess S. T., Girirajan T. P. & Mason M. D. Ultra-high resolution imaging by fluorescence photoactivation localization microscopy. Biophys. J. 91, 4258–4272 (2006). 16980368 10.1529/biophysj.106.091116 PMC1635685

[b12] Burnette D. T., Sengupta P., Dai Y., Lippincott-Schwartz J. & Kachar B. Bleaching/blinking assisted localization microscopy for superresolution imaging using standard fluorescent molecules. Proc. Natl. Acad. Sci. USA 108, 21081–21086 (2011). 22167805 10.1073/pnas.1117430109 PMC3248526

[b13] Rust M. J., Bates M. & Zhuang X. Sub-diffraction-limit imaging by stochastic optical reconstruction microscopy (STORM). Nat. Methods 3, 793–795 (2006). 16896339 10.1038/nmeth929 PMC2700296

[b14] Bates M., Huang B., Dempsey G. T. & Zhuang X. Multicolor super-resolution imaging with photo-switchable fluorescent probes. Science 317, 1749–1753 (2007). 17702910 10.1126/science.1146598 PMC2633025

[b15] van de Linde S., Heilemann M. & Sauer M. Live-cell super-resolution imaging with synthetic fluorophores. Annu. Rev. Phys. Chem. 63, 519–540 (2012). 22404589 10.1146/annurev-physchem-032811-112012

[b16] Vogelsang J. *et al.* Make them blink: probes for super-resolution microscopy. Chemphyschem 11, 2475–2490 (2010). 20632356 10.1002/cphc.201000189

[b17] Dertinger T., Colyer R., Iyer G., Weiss S. & Enderlein J. Fast, background-free, 3D super-resolution optical fluctuation imaging (SOFI). Proc. Natl. Acad. Sci. U. S. A. 106, 22287–22292 (2009). 20018714 10.1073/pnas.0907866106 PMC2799731

[b18] Dertinger T., Heilemann M., Vogel R., Sauer M. & Weiss S. Superresolution optical fluctuation imaging with organic dyes. Angew. Chem. 49, 9441–9443 (2010). 21031383 10.1002/anie.201004138 PMC3007670

[b19] Dedecker P., Mo G. C., Dertinger T. & Zhang J. Widely accessible method for superresolution fluorescence imaging of living systems. Proc. Natl. Acad. Sci. USA 109, 10909–10914 (2012). 22711840 10.1073/pnas.1204917109 PMC3390831

[b20] Cho S. *et al.* Simple super-resolution live-cell imaging based on diffusion-assisted Forster resonance energy transfer. Sci. Rep. 3, 1208 (2013). 23383376 10.1038/srep01208 PMC3563037

[b21] Goodman J. W. in Speckle phenomena in optics: theory and applications 1st edn (ed. Young Lee A.) Ch. 4, 59–140 (Roberts and Company, 2007).

[b22] Mudry E. *et al.* Structured illumination microscopy using unknown speckle patterns. Nat. Photonics 6, 312–315 (2012).

[b23] Min J. *et al.* Fluorescent microscopy beyond diffraction limits using speckle illumination and joint support recovery. Sci. Rep. 3, 2075 (2013). 23797902 10.1038/srep02075 PMC3691569

[b24] Shapiro B. Large intensity fluctuations for wave propagation in random media. Phys. Rev. Lett. 57, 2168–2171 (1986). 10033653 10.1103/PhysRevLett.57.2168

[b25] Stephen M. J. & Cwilich G. Intensity correlation functions and fluctuations in light scattered from a random medium. Phys. Rev. Lett. 59, 285–287 (1987). 10035721 10.1103/PhysRevLett.59.285

[b26] Pnini R. & Shapiro B. Fluctuations in Transmission of Waves through Disordered Slabs. Phys. Rev. B 39, 6986–6994 (1989). 10.1103/physrevb.39.6986 9947346

[b27] Garcia N., Genack A., Pnini R. & Shapiro B. Intensity correlation in waveguides. Phys. Lett. A 176, 458–461 (1993).

[b28] Feng S. C., Kane C., Lee P. A. & Stone A. D. Correlations and Fluctuations of Coherent Wave Transmission through Disordered Media. Phys. Rev. Lett. 61, 834–837 (1988). 10039442 10.1103/PhysRevLett.61.834

[b29] Freund I., Rosenbluh M. & Feng S. Memory Effects in Propagation of Optical Waves through Disordered Media. Phys. Rev. Lett. 61, 2328–2331 (1988). 10039084 10.1103/PhysRevLett.61.2328

[b30] Dertinger T., Colyer R., Vogel R., Enderlein J. & Weiss S. Achieving increased resolution and more pixels with Superresolution Optical Fluctuation Imaging (SOFI). Opt. Express 18, 18875–18885 (2010). 20940780 10.1364/OE.18.018875 PMC3072111

[b31] Geissbuehler S. *et al.* Mapping molecular statistics with balanced super-resolution optical fluctuation imaging (bSOFI). Opt. Nanosc. 1, 1–7 (2012).

[b32] Sebbah P., Hu B., Genack A. Z., Pnini R. & Shapiro B. Spatial-field correlation: the building block of mesoscopic fluctuations. Phys. Rev. Lett. 88, 123901 (2002). 11909461 10.1103/PhysRevLett.88.123901

[b33] Apostol A. & Dogariu A. Spatial correlations in the near field of random media. Phys. Rev. Lett. 91, 093901 (2003). 14525182 10.1103/PhysRevLett.91.093901

[b34] Apostol A. & Dogariu A. First- and second-order statistics of optical near fields. Opt. Lett. 29, 235–237 (2004). 14759036 10.1364/ol.29.000235

[b35] Carminati R. Subwavelength spatial correlations in near-field speckle patterns. Phys. Rev. A 81 (2010).

