# Supplementary material for: Superresolution imaging with optical fluctuation using speckle patterns illumination
Source: Sci Rep. 2015 Nov 17;5:16525. doi: 10.1038/srep16525 (PMC4648106; doi:10.1038/srep16525)
Supplement: Supplementary Information [file srep16525-s1.pdf]

Superresolution imaging with optical fluctuation using speckle patterns illumination

MinKwan Kim[1,2,†], ChungHyun Park[2, 3,†], Christophe Rodriguez[2,3], YongKeun Park[2*] and Yong-Hoon Cho[2,3*]

[1]Graduate School of Nanoscience and Technology, Korea Advanced Institute of Science and Technology, Daejeon 305-701, Republic of Korea.

[2]Department of Physics, Korea Advanced Institute of Science and Technology, Daejeon 305-701, Republic of Korea.

[3]KI for the NanoCentury, Korea Advanced Institute of Science and Technology, Daejeon 305-701, Republic of Korea.

†These authors contributed equally to this work.

*Correspondence:

Prof. YongKeun Park,

Department of Physics, Korea Advanced Institute of Science and Technology, Daejeon 305-701, Republic of Korea. Tel: (82) 42-350-2514, Email: yk.park@kaist.ac.kr

Prof. Yong-Hoon Cho

Department of Physics, Korea Advanced Institute of Science and Technology, Daejeon 305-701, Republic of Korea. Tel: (82) 42-350-2549, Email: yhc@kaist.ac.kr

A. Correlation of speckle patterns.

a. Short distance and long distance correlation of speckle patterns

Correlation of speckle patterns is critical for defining the resolution of superresolution optical fluctuation images with speckle pattern illumination (S-SOFI). This correlation has been done by Shapiro[1]. Shapiro considered the fluctuations of light scattered from a point source in a disordered medium. His results give the behavior of the correlation function at short distances (less than submicron regime, determined by the minimum value between a mean free path l and wavelength λ of incident light) but not over larger distances. To continue, long range correlation in the intensity correlation functions was derived by Stephen *et al.*[2]. From that, cumulant correlation functions of transmission in an elastic scattering medium with slab area A and thickness L, all dimensions being greater than l, is given by

$$C(\Delta\boldsymbol{r}) = C\left(\frac{l}{L}\right)^2\left(\frac{sin(k\Delta\boldsymbol{r})}{k\Delta\boldsymbol{r}}\right)^2 e^{-r/l}\ at\ \Delta\boldsymbol{r} < l,$$

$$C(\Delta\boldsymbol{r}) = \frac{27}{2k^2l^2}\left(\frac{l}{L}\right)^3\left[\frac{L}{\Delta r} - 1 + \left(\frac{\Delta r^2}{4L^2}\right)\xi(3)\right]\ at\ l < \Delta\boldsymbol{r} < L, \tag{S1}$$

where $\Delta\boldsymbol{r}$ denotes the relative position between the disordered medium and the light source, k denotes a wavevector of the light source, C denotes a numerical constant and ξ denotes a Riemann zeta function.

b. Modified correlation using a linear optics system

Following the correlation equation, the disordered medium needs to be controlled since correlation depends on r. This is a challenge for S-SOFI because it is difficult to control position in a submicron regime. However, this correlation function can be modified using a linear optics system for demagnification system, according to Goodman[3]. To simplify the linear optics system, assume that the speckle field is defined by coordinate $\boldsymbol{r}_s$ on surface of a disordered medium. Light from the speckle field then passes through 4f system (demagnification system) composed of two lenses (l_1, l_2) with focal length (f_1, f_2). With pupil positioned at the backside of l_2 (coordinate $\boldsymbol{r}_b$), speckle patterns form on the image plane (coordinate $\boldsymbol{r}_i$), as shown in Fig. S1. From this 4f system, we can derive the following equation

$$T(\boldsymbol{r}_i) = \frac{j}{M}\left\{t\left(\frac{r_i}{M}\right) \otimes PSF(\boldsymbol{r}_i)\right\} = \frac{j}{M}\iint_{-\infty}^{\infty} t\left(\frac{r_s}{M}\right) PSF(\boldsymbol{r}_i - \boldsymbol{r}_s) d^2\boldsymbol{r}_s, \tag{S2}$$

where

$$PSF(\boldsymbol{r}_i) = \iint_{-\infty}^{\infty} P(\boldsymbol{r}_b)\, exp\left(-j\frac{k}{\lambda f_2}\boldsymbol{r}_b \cdot \boldsymbol{r}_i\right) d^2\boldsymbol{r}_b,$$

$\otimes$ denotes convolution, $M = -\frac{f_2}{f_1}$ denotes magnification of the 4f system, $t(\cdot)$ denotes a speckle field on the surface of a disordered medium, $T(\cdot)$ denotes a speckle field on the imaging plane, $P(\cdot)$ denotes pupil function and $PSF(\cdot)$ denotes the point spread function of the 4f system. To derive the correlation function of speckle intensity, the speckle field should be calculated to determine speckle intensity. From this, the speckle intensity correlation is given by

$$\langle I(\boldsymbol{r}_i)I(\boldsymbol{r}_i')\rangle_{corr} = \frac{1}{M^4}\iint_{-\infty}^{\infty}\iint_{-\infty}^{\infty}\iint_{-\infty}^{\infty}\iint_{-\infty}^{\infty} \langle t\left(\frac{\boldsymbol{r}_s}{M}\right) t^*\left(\frac{\boldsymbol{r}_s'}{M}\right) t\left(\frac{\boldsymbol{r}_s''}{M}\right) t^*\left(\frac{\boldsymbol{r}_s'''}{M}\right)\rangle_{corr}\ PSF(\boldsymbol{r}_i - \boldsymbol{r}_s)PSF^*(\boldsymbol{r}_i - \boldsymbol{r}_s')$$

$$\times PSF(\boldsymbol{r}_i' - \boldsymbol{r}_s'')PSF^*(\boldsymbol{r}_i' - \boldsymbol{r}_s''')d^2\boldsymbol{r}_s d^2\boldsymbol{r}_s' d^2\boldsymbol{r}_s'' d^2\boldsymbol{r}_s''', \quad \text{(S3)}$$

where $I(\boldsymbol{r}_i) = T(\boldsymbol{r}_i)T^*(\boldsymbol{r}_i)$, and the star indicates a complex conjugate. Speckle intensity correlation can be simplified using the complex Gaussian moment theorem[1], $\langle A_1^* A_2^* A_3 A_4\rangle_{corr} = \langle A_1^* A_3\rangle_{corr} \cdot \langle A_2^* A_4\rangle_{corr} + \langle A_1^* A_4\rangle_{corr} \cdot \langle A_2^* A_3\rangle_{corr}$where A_i $(i = 1..4)$ is the speckle field; and using the approximation of the speckle field correlation[4,5], $\iint \langle t(\boldsymbol{r}_s)t(\boldsymbol{r}_s')\rangle_{corr} d^2\boldsymbol{r}_s' = \iint C(\Delta\boldsymbol{r}_s)\langle|t(\boldsymbol{r}_s)|^2\rangle d^2\boldsymbol{r}_s' \approx R\langle|t(\boldsymbol{r}_s)|^2\rangle$ where $\Delta\boldsymbol{r}_s = \boldsymbol{r}_s - \boldsymbol{r}_s'$ and $C(\cdot)$ is the correlation function. Here, equation (S1), $\langle|t(\Delta\boldsymbol{r}_s)|^2\rangle$ is the average value and depends on the geometry of the experiment, and on the thickness of the sample, and R is a proportional constant. In the case of approximation of the speckle field correlation, it should be validated that the correlation function is a very sharp function. Because the 4f system is a demagnification system in which $M = \frac{1}{M_d}$, the correlation function is rescaled as $C\left(\frac{\Delta r_s}{M}\right) = C(M_d \cdot \Delta\boldsymbol{r}_s)$. Thus, the correlation function can be considered a very sharp function. Therefore, equation (S3) becomes

$$\langle I(\boldsymbol{r}_i)I(\boldsymbol{r}_i')\rangle_{corr} = {M_d}^4 R^2 \iint_{-\infty}^{\infty}\iint_{-\infty}^{\infty}\{\langle|t(M_d\boldsymbol{r}_s)|^2\rangle\langle|t(M_d\boldsymbol{r}_s'')|^2\rangle|PSF(\boldsymbol{r}_i - \boldsymbol{r}_s)|^2|PSF(\boldsymbol{r}_i' - \boldsymbol{r}_s'')|^2$$

$$+\langle|t(M_d\boldsymbol{r}_s)|^2\rangle\langle|t(M_d\boldsymbol{r}_s'')|^2\rangle PSF(\boldsymbol{r}_i - \boldsymbol{r}_s)PSF^*(\boldsymbol{r}_i' - \boldsymbol{r}_s)PSF(\boldsymbol{r}_i - \boldsymbol{r}_s'')PSF^*(\boldsymbol{r}_i' - \boldsymbol{r}_s'')\}d^2\boldsymbol{r}_s d^2\boldsymbol{r}_s''$$

$$= {M_d}^4 R^2 \left\{\begin{matrix} \left|\iint_{-\infty}^{\infty}\langle|t(M_d\boldsymbol{r}_s)|^2\rangle\,|PSF(\boldsymbol{r}_i - \boldsymbol{r}_s)|^2 d^2\boldsymbol{r}_s\right|^2 \\ +\left|\iint_{-\infty}^{\infty}\langle|t(M_d\boldsymbol{r}_s)|^2\rangle PSF(\boldsymbol{r}_i - \boldsymbol{r}_s)PSF^*(\boldsymbol{r}_i' - \boldsymbol{r}_s)d^2\boldsymbol{r}_s\right|^2 \end{matrix}\right\}. \quad \text{(S4)}$$

Because ${M_d}^4 R^2 \iint_{-\infty}^{\infty}\langle|t(M_d\boldsymbol{r}_s)|^2\rangle\,|PSF(\boldsymbol{r}_i - \boldsymbol{r}_s)|^2 d^2\boldsymbol{r}_s = {M_d}^4 R^2 \iint_{-\infty}^{\infty}\langle|t(M_d\boldsymbol{r}_s'')|^2\rangle\,|PSF(\boldsymbol{r}_i' - \boldsymbol{r}_s'')|^2 d^2\boldsymbol{r}_s$

and because that has the same result as averaging the speckle intensity, we can substitute $\bar{I}$ which means averaging the speckle intensity. Then equation (S4) can be rewritten as

$$\langle I(\boldsymbol{r}_i)I(\boldsymbol{r}_i')\rangle_{corr} = {M_d}^4R^2\langle|t(M_d\boldsymbol{r}_s)|^2\rangle\left|\iint_{-\infty}^{\infty} PSF(\boldsymbol{r}_i-\boldsymbol{r}_s)PSF^*(\boldsymbol{r}_i'-\boldsymbol{r}_s)d^2\boldsymbol{r}_s\right|^2+\bar{I}^2. \quad \text{(S5)}$$

Because $\langle|t(M_d\boldsymbol{r}_s)|^2\rangle$ is an average value, we can take $\langle|t(M_d\boldsymbol{r}_s)|^2\rangle$ out of the integral in equation (S4). then using the PSF equation in equation (S2), we have

$$\langle I(\boldsymbol{r}_i)I(\boldsymbol{r}_i')\rangle_{corr} = {M_d}^4R^2\langle|t(M_d\boldsymbol{r}_s)|^2\rangle\left|\iint_{-\infty}^{\infty} PSF(\boldsymbol{r}_i-\boldsymbol{r}_s)PSF^*(\boldsymbol{r}_i'-\boldsymbol{r}_s)d^2\boldsymbol{r}_s\right|^2+\bar{I}^2$$

$$= {M_d}^4R^2\langle|t(M_d\boldsymbol{r}_s)|^2\rangle\left|\begin{array}{l}\iint_{-\infty}^{\infty}\iint_{-\infty}^{\infty} P(\boldsymbol{r}_b)P^*(\boldsymbol{r}_b')exp\left\{-j\frac{k}{\lambda f_2}(\boldsymbol{r}_b\cdot\boldsymbol{r}_i-\boldsymbol{r}_b'\cdot\boldsymbol{r}_i')\right\}\\ \times\left[\iint_{-\infty}^{\infty} exp\left\{-j\frac{k}{\lambda f_2}(\boldsymbol{r}_b'-\boldsymbol{r}_b)\cdot\boldsymbol{r}_s\right\}d^2\boldsymbol{r}_s\right]d^2\boldsymbol{r}_b d^2\boldsymbol{r}_b'\end{array}\right|^2+\bar{I}^2. \quad \text{(S6)}$$

In equation (S6), we can derive $\iint_{-\infty}^{\infty} exp\left\{-j\frac{k}{\lambda f_2}(\boldsymbol{r}_b'-\boldsymbol{r}_b)\cdot\boldsymbol{r}_s\right\}d^2\boldsymbol{r}_s = \frac{2\pi\lambda f_2}{k}\cdot\delta(\boldsymbol{r}_b'-\boldsymbol{r}_b)$. Substituting this result to equation (S6), then it becomes

$$\langle I(\boldsymbol{r}_i)I(\boldsymbol{r}_i')\rangle_{corr} = A\left|\iint_{-\infty}^{\infty}\iint_{-\infty}^{\infty} P(\boldsymbol{r}_b)P^*(\boldsymbol{r}_b')exp\left\{-j\frac{k}{\lambda f_2}(\boldsymbol{r}_b\cdot\boldsymbol{r}_i-\boldsymbol{r}_b'\cdot\boldsymbol{r}_i')\right\}\delta(\boldsymbol{r}_b'-\boldsymbol{r}_b)\,d^2\boldsymbol{r}_b d^2\boldsymbol{r}_b'\right|^2$$

$$= A\left|\iint_{-\infty}^{\infty}|P(\boldsymbol{r}_b)|^2exp\left\{-j\frac{k}{\lambda f_2}(\Delta\boldsymbol{r}_i\cdot\boldsymbol{r}_b)\right\}d^2\boldsymbol{r}_b\right|^2+\bar{I}^2, \quad \text{(S7)}$$

where $\Delta\boldsymbol{r}_i = \boldsymbol{r}_i - \boldsymbol{r}_i'$ and $A = \frac{2\pi\lambda f_2}{k}{M_d}^4R^2\langle|t(M_d\boldsymbol{r}_s)|^2\rangle$. This result, $\iint_{-\infty}^{\infty}|P(\boldsymbol{r}_b)|^2exp\left\{-j\frac{k}{\lambda f_2}(\Delta\boldsymbol{r}_i\cdot\boldsymbol{r}_b)\right\}d^2\boldsymbol{r}_b$, in equation (S7) is entirely similar to the van Cittert-Zernike theorem of classical coherence theory[6]. From this result in (S7) and the van Cittert-Zernike theorem, the speckle intensity correlation is determined on the image plane by Fourier transform of the intensity distribution across the lens pupil. Note that Fourier transform of the intensity distribution across the lens pupil is usually defined as a point spread function. Therefore, we can re-write equation (S7) as equation (2) of the main text,

$$C_m(\Delta\boldsymbol{r}_i) = A|PSF(\Delta\boldsymbol{r}_i)|^2, \quad \text{(S8)}$$

where $C_m(\Delta\boldsymbol{r}_i) = \langle I(\boldsymbol{r}_i)I(\boldsymbol{r}_i')\rangle_{corr} - \bar{I}^2 = \langle(I(\boldsymbol{r}_i)-\bar{I})(I(\boldsymbol{r}_i')-\bar{I})\rangle_{corr}$, $\bar{I}$ is the mean value of $I(\cdot)$.

c. Correlation from real speckle.

From equation (S8), we can clearly define the speckle intensity correlation modified by the linear optics

(demagnification) system. To compare this result with the experiment result, we measured speckle patterns using the experimental setup combined with disordered medium and motorize stage, to generate speckle patterns and their correlation, as shown in Fig. S2a. To prevent loss of speckle pattern spatial frequency information, an objective lens with a greater numerical aperture (NA = 0.8) compared to that of the illumination objective lens (NA = 0.5), was used as the collection objective lens. The speckle pattern correlation was calculated and averaged from 100 frames of the measured speckle patterns shown in Fig. S2b. As a result, the line profile (red dot) from the correlation calculated using the measured speckle pattern is well matched with the line profile (black line) calculated using the diffraction-limited point spread function shown in Fig. S2c. This result shows that the speckle correlation modified by the linear optics system is well described by equation (S7).

B. Analytic model

a. Mathematical calculation

Speckle patterns illumination can induce fluctuations of fluorophores, and these fluctuations can be described mathematically by

$$F(\boldsymbol{r},t) = \sum_{k=1}^{N} U(\boldsymbol{r}-\boldsymbol{r_k})\varepsilon_k S(\boldsymbol{r_k},t), \tag{S9}$$

Where $U(\cdot)$ denotes the diffraction-limited point spread function in case of incoherent source, ε_k denotes molecular brightness, and $S(\boldsymbol{r_k},t)$ represents induced-fluctuation of fluorophores using the speckle pattern at position $\boldsymbol{r_k}$ at time t as shown in equation (1) in the main text. To obtain the S-SOFI equation, we apply second order correlation, then equation (S9) becomes

$$\langle \delta F(\boldsymbol{r},t)\, \delta F(\boldsymbol{r},t+\tau)\rangle_{corr} = \langle \textstyle\sum_{j,k=1}^{N} U(\boldsymbol{r}-\boldsymbol{r_j})\, U(\boldsymbol{r}-\boldsymbol{r_k})\varepsilon_j\varepsilon_k \delta S(\boldsymbol{r_j},t)\delta S(\boldsymbol{r_k},t+\tau)\rangle_{corr}$$

$$= \sum_{j,k=1}^{N} U(\boldsymbol{r}-\boldsymbol{r_j})\, U(\boldsymbol{r}-\boldsymbol{r_k})\varepsilon_j\varepsilon_k \langle \delta S(\boldsymbol{r_j},t)\delta S(\boldsymbol{r_k},t+\tau)\rangle_{corr}, \tag{S10}$$

where $\delta F(\cdot) = F(\cdot) - \bar{F}$ and τ is the time lag between frames. Since $s_k(\boldsymbol{r_k}(t))$ is induced-fluctuation using a speckle pattern, the correlation term, $\langle \delta S(\boldsymbol{r_j},t)\delta S(\boldsymbol{r_k},t+\tau)\rangle_{corr}$, is decided by the speckle correlation. However, the original SOFI equation needs two types of correlation: cross-correlation $(j \neq k)$ and auto-correlation $(j = k)$. Regarding the original SOFI, since the blinking signals of a fluorophore has no cross-correlation with the blinking signals of other fluorophores, this is not considered, and amounts to a major assumption of SOFI. In contrast, for S-SOFI, since the fluctuation spot of a speckle pattern has limited size, we

have to consider cross-correlation of fluctuation signals from fluorophores in the same speckle spot. To do this, we need to consider an equation including both types of correlation in the analytic model used for calculation of S-SOFI. In order to simplify this problem, we expand the spot size of the incident laser toward a diffuser. Then, because a speckle pattern made from the large scale spot of an incident laser can ignore small dephasing fluctuations, the speckle pattern just shifts laterally as the diffuser is moved incrementally by the motorized stage (Fig. S3). Therefore, since the speckle pattern shifts laterally with small dephasing, the correlation function in equation (S8) can be used not only for auto-correlation, but also for cross-correlation. Substituting equation (S8) into equation (S10), we need to consider the step size of the motorized stage since the speckle pattern shifts laterally following the motorized stage; then equation (S10) becomes

$$G_2 = \sum_{j,k}^{N} U(\boldsymbol{r}-\boldsymbol{r_j})U(\boldsymbol{r}-\boldsymbol{r_k})\varepsilon_j\varepsilon_k C_m\left(\boldsymbol{r_k}-\boldsymbol{r_j}-\frac{\boldsymbol{D}_m(\tau)}{M_d}\right)$$

$$= \sum_{j,k}^{N} U(\boldsymbol{r}-\boldsymbol{r_j})U(\boldsymbol{r}-\boldsymbol{r_k})\varepsilon_j\varepsilon_k C_m\left(\boldsymbol{r_k}-\boldsymbol{r_j}-\boldsymbol{D}(\tau)\right), \quad \text{(S11)}$$

where $\boldsymbol{D}_m(\tau)$ is step size of the motorized stage and $\boldsymbol{D}(\tau)$ is rescaled $\boldsymbol{D}_m(\tau)$ according to the optical magnification. Because $\boldsymbol{D}(\tau)$ is a negligible distance compared with the FWHM of the intensity correlation function C_m, we can ignore $\boldsymbol{D}(\tau)$, and get the equation (3) in the main text from equation (S11).

$$G_2 = \sum_{j,k}^{N} U(\boldsymbol{r}-\boldsymbol{r_j})U(\boldsymbol{r}-\boldsymbol{r_k})\varepsilon_j\varepsilon_k C_m(\boldsymbol{r_k}-\boldsymbol{r_j}), \quad \text{(S12)}$$

b. Simulation of virtual sample containing two fluorophores

To define a resolution enhancement of S-SOFI, we consider two fluorophores with molecular brightness $\varepsilon_j, \varepsilon_k$ = 1 apart from each other with distance $\boldsymbol{a}$, as shown in Fig. S4a. To calculate using the analytic model, we define a fluorophore position as an expressed vector in the form $\boldsymbol{r}-\boldsymbol{r}_1 = x\hat{x} + y\hat{y}$ and $\boldsymbol{r}-\boldsymbol{r}_2 = (x-a)\hat{x} + y\hat{y}$, such that equation (S12) becomes

$$G_2 = \sum_{j,k}^{2} U(\boldsymbol{r}-\boldsymbol{r_j})U(\boldsymbol{r}-\boldsymbol{r_k})C_m(\boldsymbol{r_k}-\boldsymbol{r_j})$$

$$= U(\boldsymbol{r}-\boldsymbol{r}_1)U(\boldsymbol{r}-\boldsymbol{r}_1) + U(\boldsymbol{r}-\boldsymbol{r}_2)U(\boldsymbol{r}-\boldsymbol{r}_2) + U(\boldsymbol{r}-\boldsymbol{r}_1)U(\boldsymbol{r}-\boldsymbol{r}_2)$$

$$\times \{C_m(\boldsymbol{r}_1-\boldsymbol{r}_2) + C_m(\boldsymbol{r}_2-\boldsymbol{r}_1)\}. \quad \text{(S13)}$$

Since the measured image of a fluorophore is an incoherent image, we can use an incoherent PSF defined

modulus square of the coherent PSF, which can be approximated by a Gaussian function in the form $U(\boldsymbol{r}) = exp\left(-\frac{r^2}{w^2}\right) = exp\left(-\frac{(x^2+y^2)}{w^2}\right)$. Using the Gaussian function form in the PSF, we have

$$G_2 = exp\left(\frac{-2(x^2+y^2)}{w^2}\right) + exp\left(\frac{-2((x-a)^2+y^2)}{w^2}\right) + 2exp\left(\frac{-2\left(\left(x-\frac{a}{2}\right)^2+y^2\right)}{w^2}\right) exp\left(\frac{-3a^2}{2w^2}\right)$$

$$= exp\left(\frac{-(x^2+y^2)}{\left(w/\sqrt{2}\right)^2}\right) + exp\left(\frac{-((x-a)^2+y^2)}{\left(w/\sqrt{2}\right)^2}\right) + 2exp\left(\frac{-\left(\left(x-\frac{a}{2}\right)^2+y^2\right)}{\left(w/\sqrt{2}\right)^2}\right) exp\left(\frac{-3a^2}{2w^2}\right), \quad \text{(S14)}$$

where w is the full width at half maximum of the Gaussian function, which corresponds to $\frac{\lambda}{2NA}$ in conventional microscopy, λ is the wavelength of the detection signal. As shown in equation (S14), the width of Gaussian form PSF is reduced by factor $\sqrt{2}$ since PSF is squared according to the SOFI algorithm. Notice that the final term in equation (S14) is regarded as the contribution of a virtual fluorophore made by cross-correlation of the original two fluorophores at their center. This term affects the resolution enhancement of S-SOFI. Using this analytic model equation (S14), we can get the analytically simulated image in Fig. S4b, where $\lambda = 532\ nm$, $NA = 0.5$, and $a = 532\ nm$. We can check the resolution enhancement using the analytic model, and the intensity peak ratio of the Abbe limit. Therefore, the resolution enhancement of S-SOFI is 1.3× that of the resolution of conventional microscopy when $\lambda = 532\ nm$, and $NA = 0.5$.

C. Fourier reweighting method

The Fourier reweighting (FRW) method was introduced by Dertinger *et al.* to improve the resolution enhancement of SOFI[7]. According to the FRW method, use of a simple reweighting of the optical transfer function (OTF), the resolution enhancement of SOFI can be improved even more (e.g., $\sqrt{2} \rightarrow 2$). Specifically, the FRW method can improve the resolution of SOFI by replacing the 2-fold PSF with 4-fold PSF using the reweighting factor in Fourier space. In order to calculate the resolution enhancement of S-SOFI with FRW, we also apply FRW method to the two fluorophores analytic model as in the following step. First, Fourier transform is applied equation (14) as follows,

$$\tilde{F}\{G_2\} = \widetilde{G_2} = \tilde{F}\{U^2(\boldsymbol{r})\} + \tilde{F}\{U^2(\boldsymbol{r} - a\hat{x})\} + \tilde{F}\left\{2 \cdot U^2\left(\boldsymbol{r} - \frac{a}{2}\hat{x}\right) \cdot exp\left(\frac{-3a^2}{2w^2}\right)\right\}$$

$$= \widetilde{U}(\boldsymbol{k}) \otimes \widetilde{U}(\boldsymbol{k}) + \left\{\widetilde{U}(\boldsymbol{k}) \otimes \widetilde{U}(\boldsymbol{k})\right\} \cdot exp(-ia\boldsymbol{k} \cdot \hat{x}) + \left\{\widetilde{U}(\boldsymbol{k}) \otimes \widetilde{U}(\boldsymbol{k})\right\} \cdot exp\left(-\frac{ia\boldsymbol{k}\cdot\hat{x}}{2}\right) \cdot B, \quad \text{(S15)}$$

where $\tilde{F}$ denotes the Fourier transform, $\otimes$ denotes convolution, $\widetilde{G_2}$ represents the Fourier transform of G_2, $\tilde{U}$ represents the OTF (Fourier transformed U), and $\boldsymbol{k}$ denotes the spatial frequency vector and B=$2exp\left(\frac{-3a^2}{2w^2}\right)$.

Next, equation (S15) is multiplied using a Fourier reweighting factor defined as $w = \frac{\tilde{U}(\boldsymbol{k}/2)}{\tilde{U}(\boldsymbol{k})\otimes\tilde{U}(\boldsymbol{k})+\alpha}$, where $\alpha \ll 1$ denotes a damping factor to prevent division by close-to-zero numbers, and then equation (S15) becomes

$$\widetilde{G_2} \cong \tilde{U}(\boldsymbol{k}/2) + \tilde{U}(\boldsymbol{k}/2)\cdot exp(-ia\boldsymbol{k}\cdot\hat{x}) + \tilde{U}(\boldsymbol{k}/2)\cdot exp\left(-\frac{ia\boldsymbol{k}\cdot\hat{x}}{2}\right)\cdot B. \quad \text{(S16)}$$

And $\boldsymbol{k}$ is substituted by $\boldsymbol{k}' = \frac{\boldsymbol{k}}{2}$. Then equation (S16) can be rewritten as

$$\widetilde{G_2} \cong \tilde{U}(\boldsymbol{k}') + \tilde{U}(\boldsymbol{k}')\cdot exp(-i2a\boldsymbol{k}'\cdot\hat{x}) + \tilde{U}(\boldsymbol{k}')\cdot exp(-ia\boldsymbol{k}'\cdot\hat{x})\cdot B. \quad \text{(S17)}$$

Note that $\tilde{U}(\boldsymbol{k}/2)$ is the Fourier transform of $U(2\boldsymbol{r}) = exp\left(-\frac{4\boldsymbol{r}^2}{w^2}\right)$. Third, the inverse Fourier transform is applied to equation (S17), and then equation (S17) is given by

$$\tilde{F}^{-1}\{\widetilde{G_2}\} = G_2 \cong \tilde{F}^{-1}\{\tilde{U}(\boldsymbol{k}')\} + \tilde{F}^{-1}\{\tilde{U}(\boldsymbol{k}')\cdot exp(-i2a\boldsymbol{k}'\cdot\hat{x})\} + \tilde{F}^{-1}\{\tilde{U}(\boldsymbol{k}')\cdot exp(-ia\boldsymbol{k}'\cdot\hat{x})\cdot B\}$$

$$=U(\boldsymbol{r}') + U(\boldsymbol{r}' - 2a\hat{x}) + U(\boldsymbol{r}' - a\hat{x})\cdot B. \quad \text{(S18)}$$

Because $\boldsymbol{k}'$ is $\frac{\boldsymbol{k}}{2}$, $\boldsymbol{r}'$ can be considered$2\boldsymbol{r}$. Finally, using this relation, the FRW analytic model is gotten as

$$G_2 \cong U(2\boldsymbol{r}) + U(2\boldsymbol{r} - 2a\hat{x}) + U(2\boldsymbol{r} - a\hat{x})\cdot B$$

$$= U(2\boldsymbol{r}) + U(2(\boldsymbol{r} - a\hat{x})) + U\left(2\left(\boldsymbol{r} - \frac{a}{2}\hat{x}\right)\right)\cdot B,$$

$$= exp\left(\frac{-4(x^2+y^2)}{w^2}\right) + exp\left(\frac{-4((x-a)^2+y^2)}{w^2}\right) + 2exp\left(\frac{-4\left(\left(x-\frac{a}{2}\right)^2+y^2\right)}{w^2}\right)exp\left(\frac{-3a^2}{2w^2}\right)$$

$$= exp\left(\frac{-(x^2+y^2)}{(w/2)^2}\right) + exp\left(\frac{-((x-a)^2+y^2)}{(w/2)^2}\right) + 2exp\left(\frac{-\left(\left(x-\frac{a}{2}\right)^2+y^2\right)}{(w/2)^2}\right)exp\left(\frac{-3a^2}{2w^2}\right). \quad \text{(S19)}$$

Using this equation, we can get simulated images from the two fluorophores analytic model as shown in Fig. S4c. The resolution enhancement of S-SOFI with FRW was then evaluated using modeling and the criterion of the Abbe limit, to determine that the resolution enhancement was 1.6× compared with the resolution of

conventional microscopy when $\lambda = 532\ nm$, and $NA = 0.5$.

D. Resolution enhancement of S-SOFI with step size of the motorize stage

In manuscript of experiment, $\boldsymbol{D}(\tau)$ is relatively large value and thus cannot be neglected (e.g., when the step size of motorized stage = 6 μm, then the $\boldsymbol{D}(\tau)$ = 120nm in the nanopattern; then the step size of the motorized stage = 2 μm and 7 μm, then the $\boldsymbol{D}(\tau)$ = 200 nm and 70 nm in a biological sample). Thus, we should consider effect of $\boldsymbol{D}(\tau)$ in S-SOFI processing. In order to estimate the $\boldsymbol{D}(\tau)$ effect on the S-SOFI resolution, we needed to revisit the two fluorophore model used in section B-b. Using the two fluorophore model, the S-SOFI equation with $\boldsymbol{D}(\tau)$ is mathematically expressed as

$$G_2 = \sum_{j,k}^{2} U(r - r_j)U(r - r_k)\varepsilon_j\varepsilon_k C_m\left(r_k - r_j - \boldsymbol{D}(\tau)\right)$$

$$= exp\left(\frac{-2(x^2+y^2)}{w^2}\right) + exp\left(\frac{-2((x-a)^2+y^2)}{w^2}\right) + exp\left(\frac{-2\left(\left(x-\frac{a}{2}\right)^2+y^2\right)}{w^2}\right)exp\left(\frac{-a^2}{2w^2}\right)$$

$$\times\left\{exp\left(\frac{-(a+d)^2}{w^2}\right) + exp\left(\frac{-(a-d)^2}{w^2}\right)\right\}. \quad \text{(S20)}$$

Here, the cross correlation term affected by $\boldsymbol{D}(\tau)$ should be considered to calculate resolution enhancement of S-SOFI. Additionally, it is main factor that prevents resolution enhancement of S-SOFI approach to the root of cumulant order. Using $exp\left(\frac{-(a+d)^2}{w^2}\right) + exp\left(\frac{-(a-d)^2}{w^2}\right) = 2exp\left(-(a^2+d^2)\right)cosh\left(\frac{2ad}{w^2}\right)$ relation, equation (S20) can be rewritten as

$$G_2 = exp\left(\frac{-d^2}{w^2}\right)\left\{exp\left(\frac{-2(x^2+y^2)}{w^2}\right) + exp\left(\frac{-2((x-a)^2+y^2)}{w^2}\right) + 2exp\left(\frac{-2\left(\left(x-\frac{a}{2}\right)^2+y^2\right)}{w^2}\right)exp\left(\frac{-3a^2}{2w^2}\right)cosh\left(\frac{2ad}{w^2}\right)\right\}, \quad \text{(S21)}$$

Comparing with equation (S14), equation (S21) has additional two terms which are $exp\left(\frac{-d^2}{w^2}\right)$, $cosh\left(\frac{2ad}{w^2}\right)$. The first term, $exp\left(\frac{-d^2}{w^2}\right)$, affects the entire intensity of reconstructed image when using S-SOFI. However, the second term $cosh\left(\frac{2ad}{w^2}\right)$ affects the resolution enhancement of S-SOFI. To estimate the effect of the second term, the distance of two fluorophores a is fixed as $\frac{w}{1.3}$ and $\frac{w}{1.6}$ when considering resolution enhancement factor of S-SOFI. Then, the graph of $cosh\left(\frac{2ad}{w^2}\right)$ is obtained, as shown in Fig. S5. This graph shows that the

resolution enhancement of S-SOFI is degrade less than 10% by the $\boldsymbol{D}(\tau)$ effect, when d is less than $\frac{1}{3}w$. Moreover, the graphs with the value a/w varied do not show a significance difference when d is fixed 0 and $\frac{1}{3}w$ respectively. To check this effect more clearly, we did a two fluorophores simulation based on equation (S21). As shown in Fig. S6, the degradation of the resolution is negligible compare to reconstructed image without $\boldsymbol{D}(\tau)$ effect. From these results, we can conclude that $\boldsymbol{D}(\tau)$ does not affect the resolution enhancement of S-SOFI within $\frac{1}{3}w$ regime. Moreover, $\boldsymbol{D}(\tau)$ is less than $\frac{1}{3}w$ ($\boldsymbol{D}(\tau) = 120\,nm < 532\,nm$ in the nanopattern; $\boldsymbol{D}(\tau) = 200\,nm < 665\,nm$ and $70\,nm < 217\,nm$ in the biological sample.) in our experiment of manuscript, results of experiment well described about resolution of S-SOFI.

E. Temporal correlation length control

The temporal correlation length using intrinsic blinking of fluorophores cannot be controlled using external methods in other superresolution methods since it is already determined by intrinsic properties of the fluorophores. To control this correlation, chemical treatment[8,9] of the fluorophores is required. However, temporal correlation in S-SOFI can be changed by controlling random patterns of illumination that can induce blinking of fluorophores. In this paper, speckle patterns were employed as random patterns, and temporal correlation made by converting spatial correlation of the speckle patterns into temporal correlation using the motorized stage. Therefore, temporal correlation could be controlled by adjusting the step size of the motorized stage. As shown in Fig. S5a, the temporal correlation length is reduced with increasing step size of the motorized stage. Since the number of image frames needed for superresolution imaging depends on the temporal correlation length, it could also be controlled by adjusting the step size of the motorized stage. However, as the step size increases, the rescaled step size $\boldsymbol{D}(\tau)$ is no longer small enough to be ignored, as shown in equation (S11). Thus, $\boldsymbol{D}(\tau)$ influences the resolution of S-SOFI and distortion occurs during resolution enhancement in the superresolution image. To remove this effect, zero is chosen as time lag τ in equation (S10) when images are analyzed using the SOFI algorithm. A similar method already used in previous work to reduce the number of image frames needed for SOFI[10,11]. Then, equation (S10) can be rewritten as

$$\langle \delta F(\boldsymbol{r},t)\, \delta F(\boldsymbol{r},t)\rangle_{corr} = \sum_{j,k=1}^{N} U(\boldsymbol{r}-\boldsymbol{r_j})\, U(\boldsymbol{r}-\boldsymbol{r_k})\varepsilon_j\varepsilon_k \langle \delta S(\boldsymbol{r_j},t)\delta S(\boldsymbol{r_k},t)\rangle_{corr}$$

$$= \sum_{j,k}^{N} U(\boldsymbol{r}-\boldsymbol{r_j})U(\boldsymbol{r}-\boldsymbol{r_k})\varepsilon_j\varepsilon_k C_m(\boldsymbol{r_k}-\boldsymbol{r_j}). \quad \text{(S22)}$$

We can get the same equation shown in equation (S12) since $\boldsymbol{D}(\tau = 0)$ is zero. Therefore, the number of image

frames needed for S-SOFI can be reduced using control of the temporal correlation length, and by applying equation (S20) without distortion of resolution enhancement. To demonstrate this, 350 images (exposure time = 0.1 s) from the biological sample (motorized stage step = 4 μm) illuminated by speckle patterns, and analyzed using SOFI and the Fourier reweighting method. As a result, we were able to get similar results using fewer image frames, as shown in Fig. S7 b-c. Moreover, this control of temporal correlation and use of equation (S20) provides not only that a reduced image frames with increasing step size of the motorized stage, but also that the random speckle patterns can be used for S-SOFI without strict control of the motorized stage. However, the number of image frames for S-SOFI cannot be reduced infinitely with increasing step size because the signal to noise ratio (SNR) decreases with the number of image frames as shown in Fig. S6. Thus, the number of image frames can be reduced by adjusting step size within the range needed to acquire enough image frames to satisfy the target SNR. Another, possible factor in the reduction of the number of image frames is the distortion effect of the speckle pattern may be used to increase randomness of the speckle pattern, unlike as shown in Fig. S3. When we apply this distortion effect to S-SOFI, it is expected that the number of image frames will be reduced even more.

F. Uniform illumination using speckle pattern

Each frame of the speckle pattern shows nonuniform illumination characteristics, as shown in Fig. S3. To achieve a uniform illumination using the average of the speckle patterns, a sufficient number of frames of the speckle patterns are required. To quantify this, we used the concept of the intensity contrast of the speckle pattern. Theoretically, when a diffuser is continuously moving, the speckle pattern contrast is determined by following equation[12]:

$$C = \sqrt{\frac{\tau_c}{T}} = 0.52\sqrt{\frac{w}{vT}}, \text{ when } T \gg \tau_c \text{ (Circular pupil case),} \tag{S23}$$

where C is the contrast of speckle pattern, w is the full with half maximum of the point spread function, v is the velocity of the diffuser, T is the total exposure time and τ_c is the correlation time of the speckle pattern. Similarly, because S-SOFI uses varying speckle patterns over time, the averaging process of the speckle patterns can be regarded as a continuously moving diffuser. To apply the moving diffuser equation for S-SOFI, the equation (S23) is modified as follows:

$$C = 0.52\sqrt{\frac{w}{DM}}, \text{ when } D < \frac{w}{4} \text{ (Circular pupil case and continuous regime),}$$

$$C = \sqrt{\frac{1}{M}}, \text{ when } D < \frac{w}{4} \text{ (General case and discontinuous regime),} \quad \text{(S24)}$$

Where D denotes the demagnified step size of the motorized stage and M denotes the number of frames. Using these equations, contrast of the averaged speckle pattern can be calculated as shown in Fig. S9. To distinguish two objects from one another with half the wavelength, the variation in image contrast should not exceed 10% based on Abbe’s criterion. From this result, a sufficient number of frames of the speckle patterns can be estimated. Moreover, the results of the averaged image from biological sample exhibit similar contrast over estimated number of frames using contrast equation, as shown in Fig. S10. However, some results from the biological sample show clearer image than the predicted one although they used images that had less than the estimated number of frames. This can be attributed to the overestimation of the speckle contrast equation because we only took into account simple parameters of the speckle patterns for the calculation. Nevertheless, the equations used well describe the relationship between the uniformity and the speckle contrast.

References

1 Shapiro, B. Large intensity fluctuations for wave propagation in random media. *Phys. Rev. Lett.* **57**, 2168-2171 (1986).

2 Stephen, M. J. & Cwilich, G. Intensity correlation functions and fluctuations in light scattered from a random medium. *Phys. Rev. Lett.* **59**, 285-287 (1987).

3 Goodman, J. W. in *Speckle phenomena in optics: theory and applications 1st edn* (ed. Lee A. Young) Ch. 4, 59-140 (Roberts and Company, 2007).

4 Pnini, R. & Shapiro, B. Fluctuations in Transmission of Waves through Disordered Slabs. *Phys. Rev. B* **39**, 6986-6994 (1989).

5 Min, J. *et al.* Fluorescent microscopy beyond diffraction limits using speckle illumination and joint support recovery. *Sci. Rep.* **3**, 2075 (2013).

6 Goodman, J. W. & Haupt, R. L. in *Statistical optics 2nd edn* (ed. Glenn Boreman) Ch. 5, 208-214 (John Wiley & Sons, 2015).

7 Dertinger, T., Colyer, R., Vogel, R., Enderlein, J. & Weiss, S. Achieving increased resolution and more pixels with Superresolution Optical Fluctuation Imaging (SOFI). *Opt. Express* **18**, 18875-18885 (2010).

8 van de Linde, S., Heilemann, M. & Sauer, M. Live-cell super-resolution imaging with synthetic fluorophores. *Annu. Rev. Phys. Chem.* **63**, 519-540 (2012).

9 Vogelsang, J. *et al.* Make them blink: probes for super-resolution microscopy. *Chemphyschem* **11**, 2475-2490 (2010).

10 Dertinger, T., Colyer, R., Iyer, G., Weiss, S. & Enderlein, J. Fast, background-free, 3D super-resolution optical fluctuation imaging (SOFI). *Proc. Natl. Acad. Sci. U. S. A.* **106**, 22287-22292 (2009).

11 Watanabe, T. M., Fukui, S., Jin, T., Fujii, F. & Yanagida, T. Real-time nanoscopy by using blinking enhanced quantum dots. *Biophys. J.* **99**, L50-52 (2010).

12 Goodman, J. W. in *Speckle phenomena in optics: theory and applications 1st edn* (ed. Lee A. Young) Ch. 5, 143-150 (Roberts and Company, 2007).

Figure legends

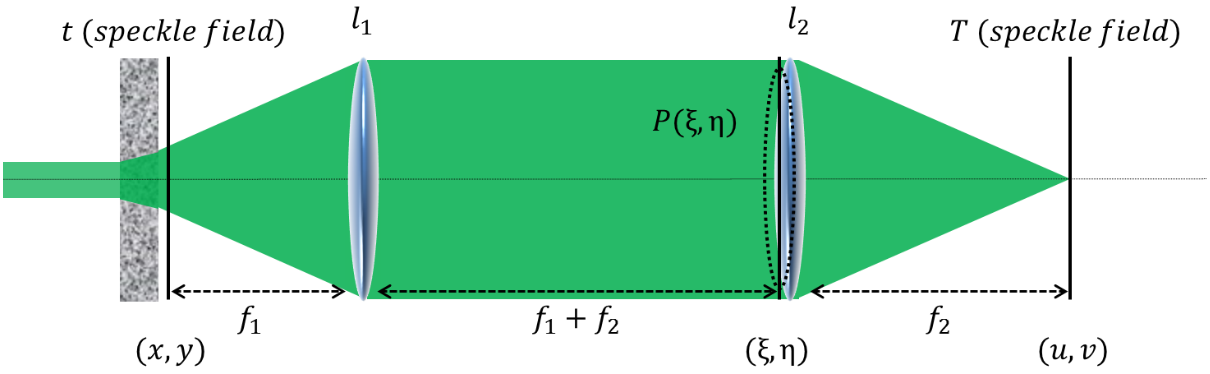

Figure S1| Modeling of 4f system for analytic calculation of speckle correlation. Here, $\boldsymbol{t}$ is the speckle field that exists on a surface, at coordinate $(\boldsymbol{x}, \boldsymbol{y})$, made from the disordered medium. This speckle field propagates and is changed to speckle field $\boldsymbol{T}$ on the object plane at coordinate $(\boldsymbol{u}, \boldsymbol{v})$ after passing through the 4f system made of lens $\boldsymbol{l_1}$ with focal length $\boldsymbol{f_1}$, lens $\boldsymbol{l_2}$ with focal length $\boldsymbol{f_2}$, and the pupil of the second lens with coordinate $(\boldsymbol{\xi}, \boldsymbol{\eta})$.

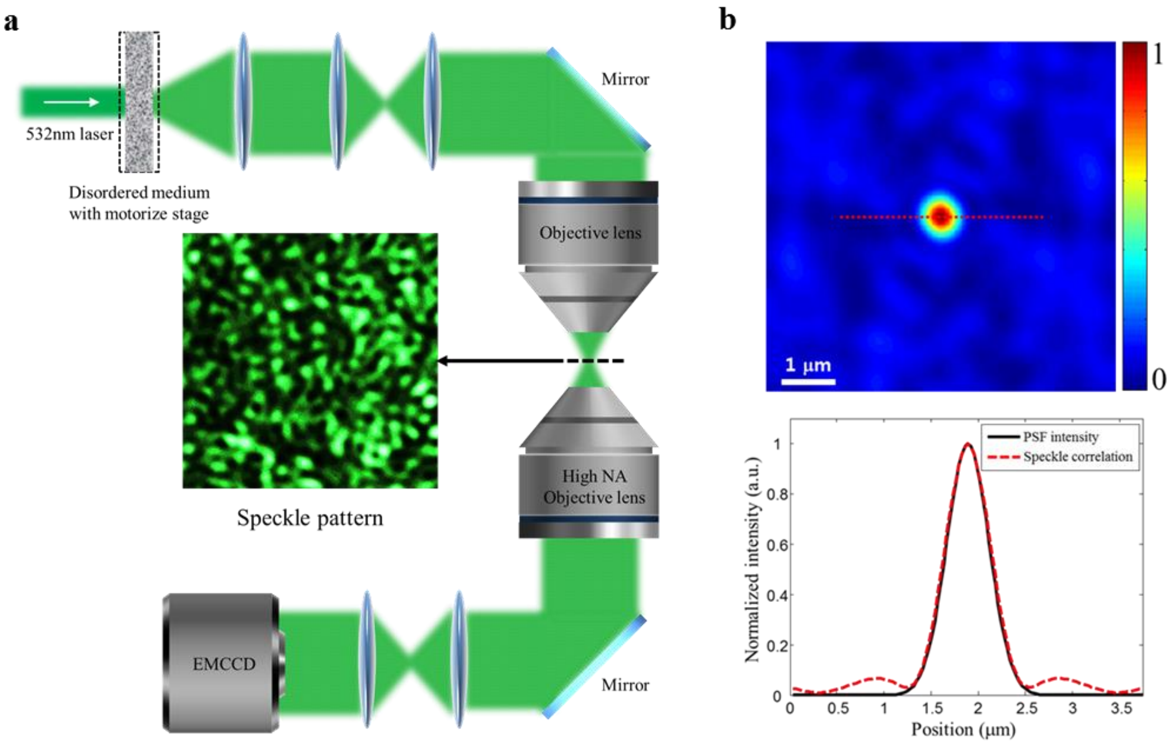

Figure S2| Experimental setup for measurement of speckle patterns and correlation from measured speckle patterns. (a) To illuminate and measure speckle patterns without a sample, an experimental setup was created using a conventional microscope (BXFM, Olympus, USA) for illumination and a conventional inverted microscope (IX71, Olympus, USA) for detection. To prevent loss of the speckle pattern spatial frequency, the detection objective lens used was of higher NA (0.8) than the illumination objective lens NA (0.5). The illuminated speckle patterns propagate through the detection scope and are measured in an electron-multiplying charge coupled device (EMCCD). (b) The upper image is a correlation image from the speckle pattern measured with the experimental setup shown in Fig. S2a. The lower image is a line profile along the red dashed line in the upper image. This line profile shows that speckle pattern correlation follows the line profile of PSF intensity from the theoretical calculation. The color bar represents normalized intensity.

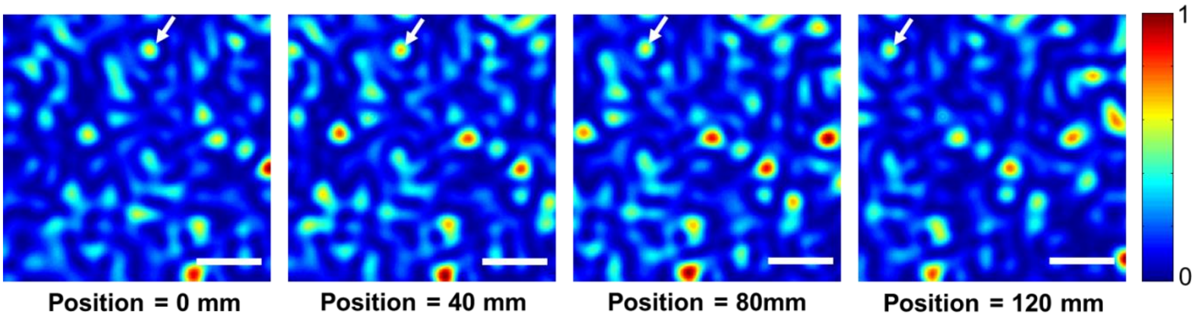

Figure S3| Lateral shift of measured speckle pattern with step size. Measured speckle pattern is laterally shifted by moving the diffuser with the motorized stage as shown by the white arrow in each part. The reason for this is that distortion of the speckle pattern caused when displacement of the diffuser is too small, compared with size of a big beam, can be ignored. The color bar represents normalized intensity. The scale bars in all figures indicate 2 μm.

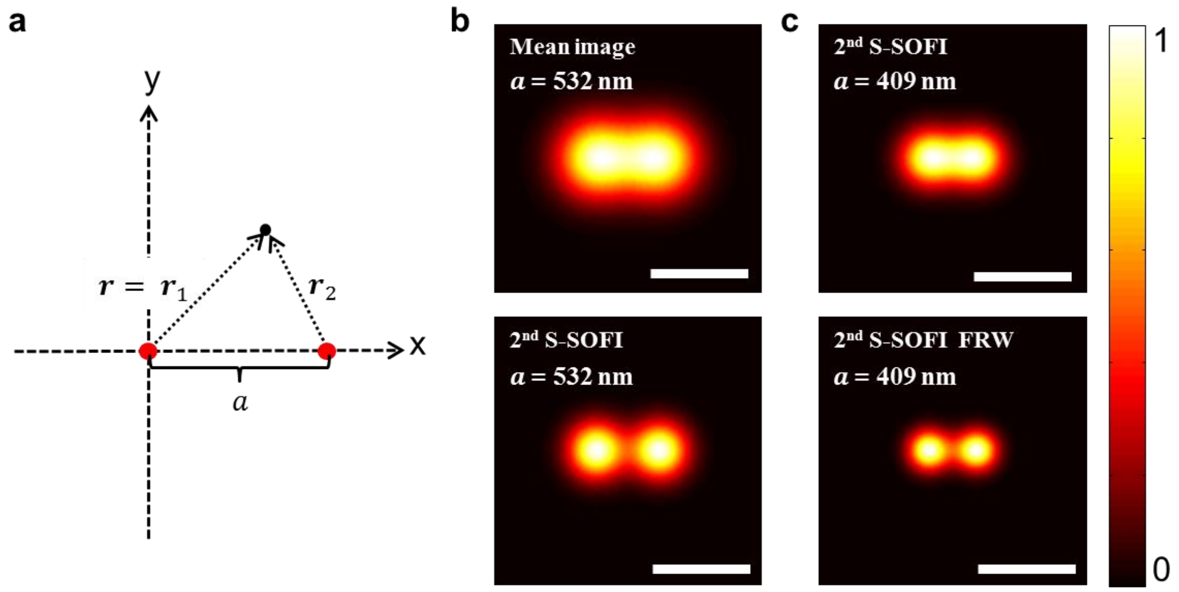

Figure S4| Two fluorophores model for simplifying analytic model and image. (a) To simplify the analytic model, the object considered included only two fluorophores separated from each other by distance $\boldsymbol{a}$. (b) The upper image is the mean image, which is equivalent to a conventional microscopy image of two fluorophores, and was calculated using the analytic model with $\boldsymbol{a} = \mathbf{532\ nm}$**.** The lower image is the 2nd S-SOFI image from the two fluorophores analytic model with $\boldsymbol{a} = \mathbf{532\ nm}$, as described in equation (S14). Note that the 2nd S-SOFI image clearly shows higher resolution than the mean image. (c) The upper image is a 2nd S-SOFI image of two fluorophores calculated using the analytic model, with $\boldsymbol{a} = \mathbf{409\ nm}$. The lower image is 2nd S-SOFI with FRW of the two fluorophores analytic model with $\boldsymbol{a} = \mathbf{409\ nm}$, as described in equation (S19). Note that the 2nd S-SOFI FRW image clearly shows higher resolution than the 2nd S-SOFI image. The color bar represents normalized intensity. The scale bars in all figures indicate 1 μm

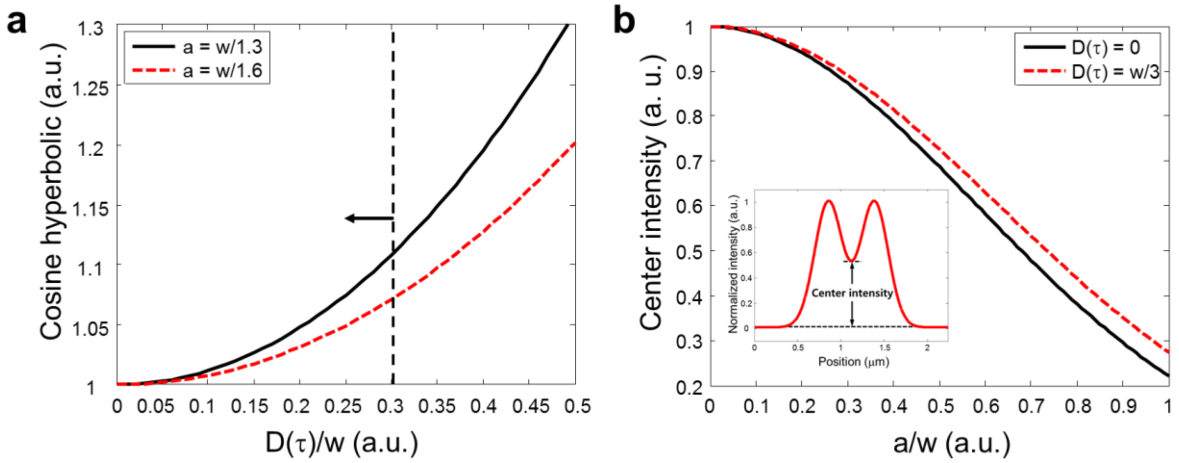

Figure S5| Graph of the second term, cosine hyperbolic, as function of D(τ)/w and a graph of the center intensity between two fluorophores due to D(τ) effect on S-SOFI resolution enhancement in the two fluorophore model. The step size of the motorized stage (D(τ)) affects the resolution enhancement of S-SOFI because D(τ) is not small enough to be neglected in this experiment. (a) To analyze the effect of D(τ) on the resolution enhancement, the second term of equation (S21) is calculated in terms of D(τ)/w with the values for the distance between two fluorophores fixed as w/1.3 and w/1.6 respectively. From this result, when D(τ) is less than ~0.3w, the resolution enhancement of S-SOFI is degraded less than 10%. This means that D(τ) does not affect the resolution of S-SOFI in this regime. (b) Additionally, when D(τ) is fixed as 0 and w/3, the center intensity defined in inset of graph is calculated by the equation (S21). The two graphs show similar results.

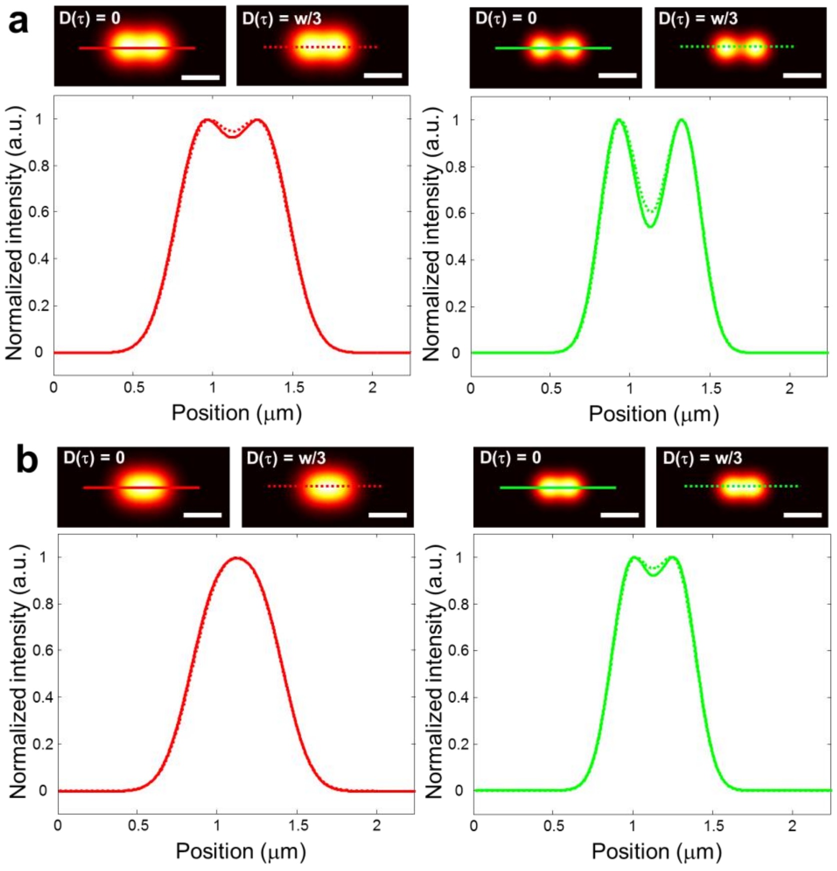

Figure S6| Simulation results with D(τ) using a virtual sample with two fluorophores. To demonstrate the relationship between the resolution of S-SOFI and D(τ), a simulation was done based on equation (S21). (a) The images show the simulation results of S-SOFI (left) and S-SOFI with fourier reweighting method (right) with D(τ) = 0 and D(τ) = w/3, when the distance *a* between the two fluorophores is w/1.3, respectively. As shown in both images, the simulation results do not have significant difference. Besides, each cross-sectional line profile taken from the figure shows similar result. (b) The same simulation results with *a* = w/1.6. When D(τ) is 0 and w/3, these results also show similar results. All the scale bars indicate 500 nm.

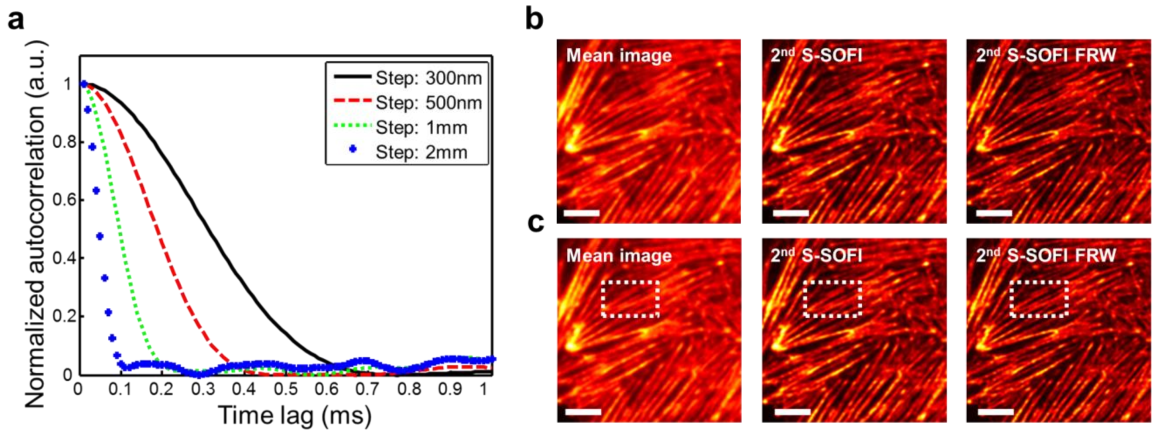

Figure S7| Temporal correlation graph with increasing step size of the motorized stage, and demonstration of S-SOFI using fewer images of the biological sample. (a) Four sets of speckle pattern images, from 1000 images of speckle patterns (exposure time = 10 ms) in each set, measured by changing the step size of the motorized stage in the experimental setup. They were analyzed using correlation analysis. The correlation graph from this analysis shows a decreasing tendency of correlation length with increasing step size. From this tendency, the length of correlation was controlled by changing the step size. (b) This images show mean image, 2nd S-SOFI and 2nd S-SOFI FRW from 700 measured images of biological sample (exposure time = 100 ms, motorized stage step = 2 μm), respectively. (c) Similarly, this images are mean image, 2nd S-SOFI and 2nd S-SOFI FRW from 350 measured images of biological sample (exposure time = 0.1 ms, motorized stage step = 4 μm), respectively. Compare b and c, these images shows almost the same images despite of the different number of image frames. From these results, we verified that the number of image frames for S-SOFI could be reduced using control of temporal correlation length. All the scale bars in all figures indicate 10 μm

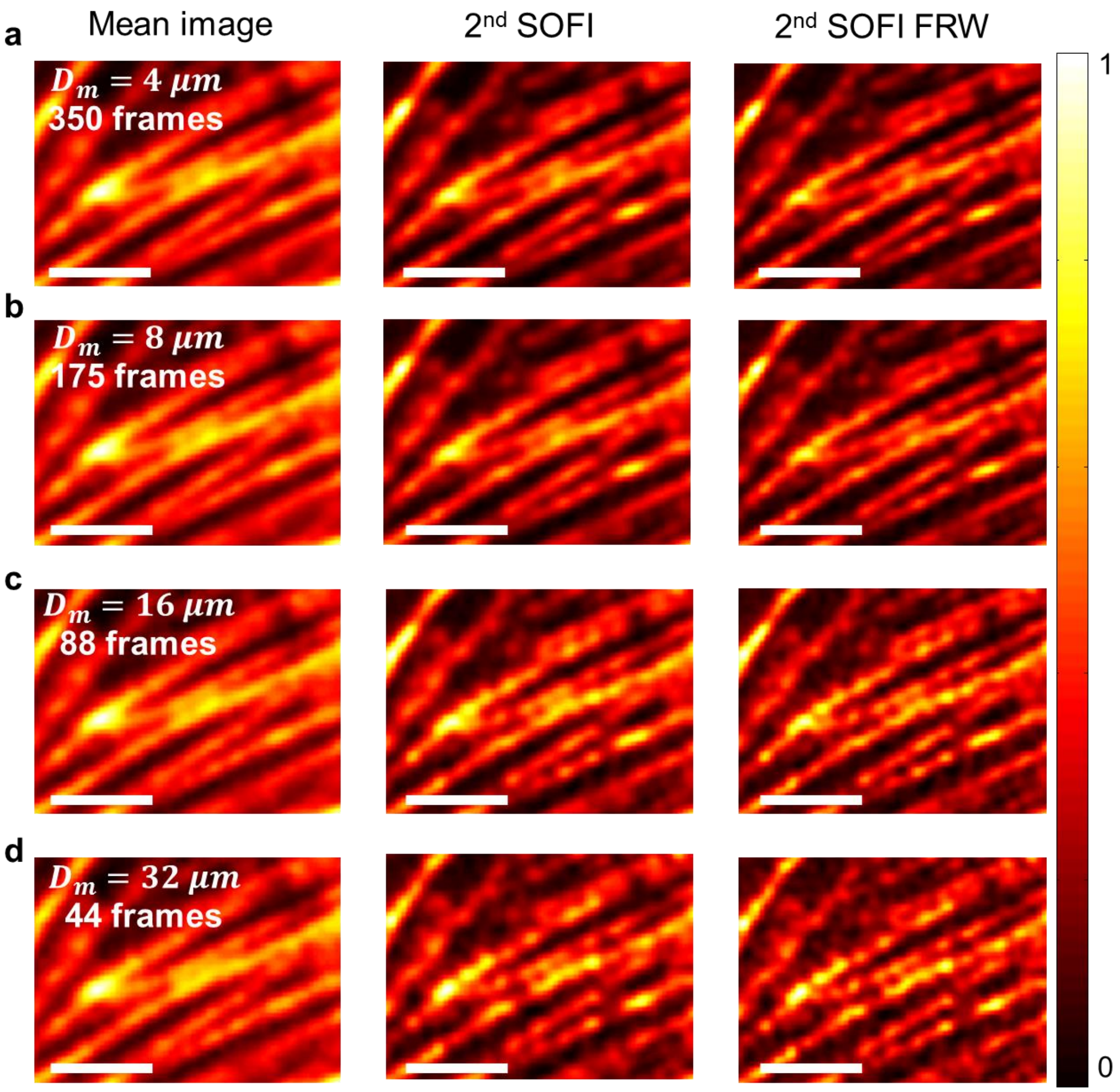

Figure S8| S-SOFI images of biological sample with increasing step size of the motorized stage. As the step size increased $\boldsymbol{D_m}$ = 4 μm, 8 μm, 16 μm, 32 μm in alphabetic sequence (exposure time = 100 ms), the biological sample was illuminated using speckle patterns in 350, 175, 88, and 44 frames, respectively. And each image was cropped from white box region of Fig. S4c. From these images, even though each mean image shows similar results, the 2nd S-SOFI and 2nd S-SOFI FRW images show results with lower signal to noise ratios (SNR) as the number of image frames decreases. Therefore, the number of image frames cannot be decreased infinitely because of the SNR problem. All the scale bars in all figures indicate 10 μm

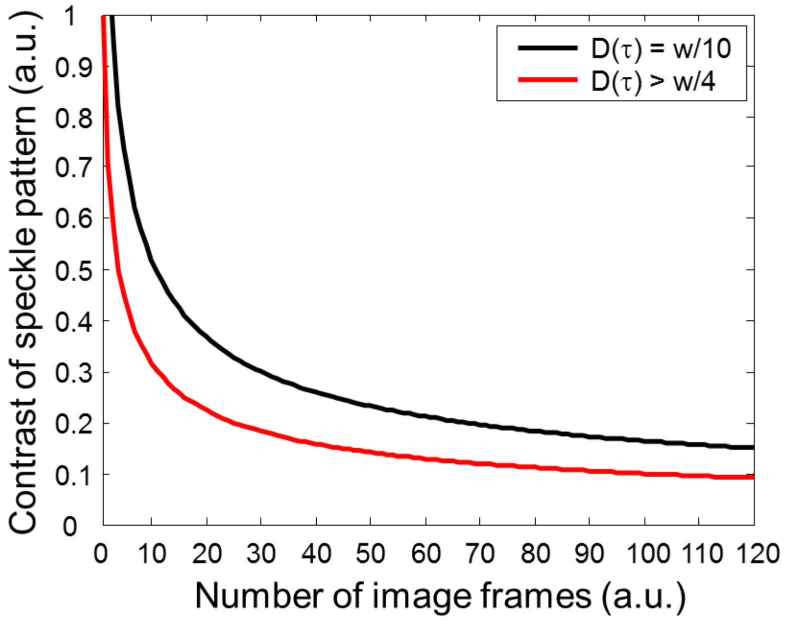

Figure S9| Graph of the speckle pattern contrast with respect to the number of image frames. The contrast of the speckle pattern was calculated in terms of the number of image frames. The black line and red lines indicate the results of the speckle pattern contrast with $D(\tau) = w/10$ and $D(\tau) > w/4$, respectively. From these results, the number of speckle pattern frames could be estimated to achieve uniform illumination.

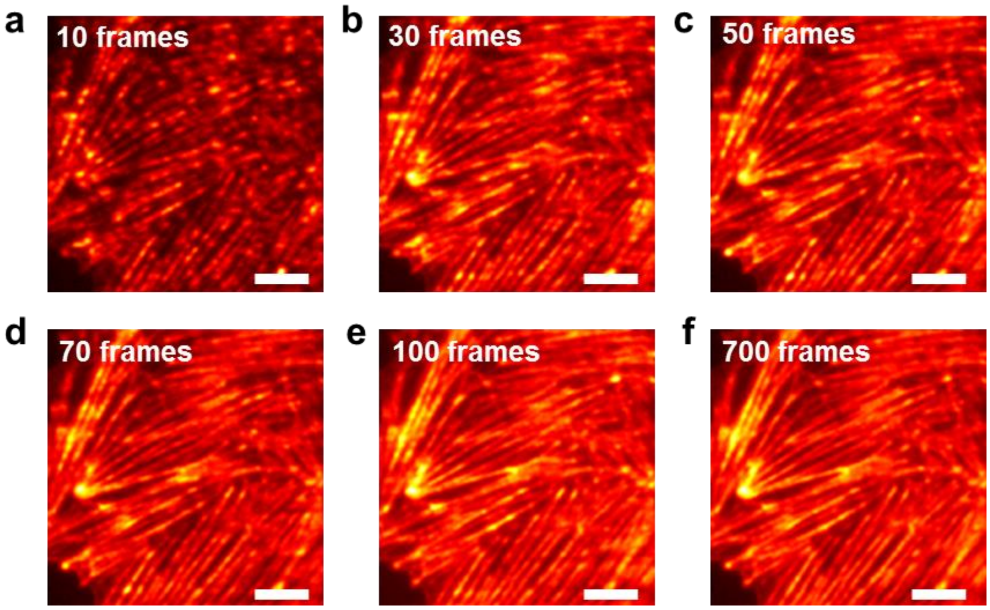

Figure S10| Averaged images of the biological sample with respect to the frame number of the speckle pattern illumination. Biological images were averaged to achieve a uniform illumination with 10, 30, 50, 70, 100, 700 frames, respectively. (a-c) Because the contrast of the speckle pattern is not sufficiently suppressed, each image shows nonuniform image. (d-f) The averaged images of the biological sample clearly show the biological structure. Moreover, they provide similar results because the number of image is sufficient to suppress the speckle pattern. All the scale bars indicate 5 μm.

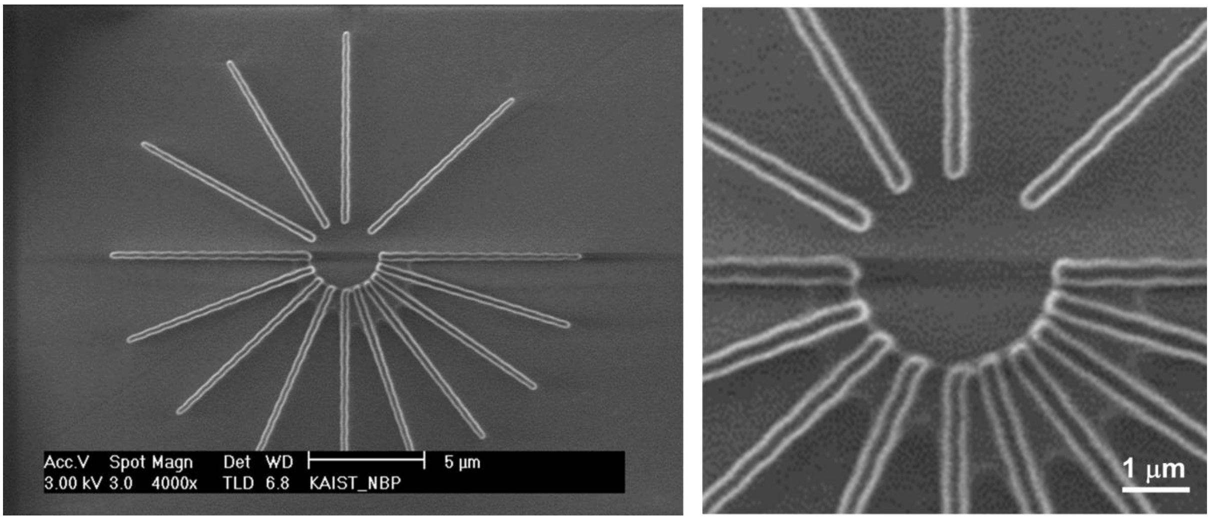

Figure S11| Scanning electron microscopy (SEM) image of the radial fluorescent nanopattern used for S-SOFI experiment.

Supplementary Movie 1: Images of the radial fluorescent nanopattern illuminated by speckle pattern.
